# Firefighters’ exposure to per-and polyfluoroalkyl substances (PFAS) as an occupational hazard: A review

**DOI:** 10.3389/fmats.2023.1143411

**Published:** 2023-03-23

**Authors:** Nur-Us-Shafa Mazumder, Md Tanjim Hossain, Fatema Tuj Jahura, Arjunsing Girase, Andrew Stephen Hall, Jingtian Lu, R. Bryan Ormond

**Affiliations:** Textile Protection and Comfort Center, Wilson College of Textiles, North Carolina State University, Raleigh, NC, United States

**Keywords:** PFAS, firefighters, cancer, turnout gear, AFFF, DWR, repellent finish, occupational hazard

## Abstract

The term “firefighter” and “cancer” have become so intertwined in the past decade that they are now nearly inseparable. Occupational exposure of firefighters to carcinogenic chemicals may increase their risk of developing different types of cancer. PFAS are one of the major classes of carcinogenic chemicals that firefighters are exposed to as occupational hazard. Elevated levels of PFAS have been observed in firefighters’ blood serum in recent studies. Possible sources of occupational exposure to PFAS include turnout gear, aqueous film-forming foam, and air and dust at both the fire scene and fire station. Preliminary discussion on PFAS includes definition, classification, and chemical structure. The review is then followed by identifying the sources of PFAS that firefighters may encounter as an occupational hazard. The structural properties of the PFAS used in identified sources, their degradation, and exposure pathways are reviewed. The elevated level of PFAS in the blood serum and how this might associate with an increased risk of cancer is discussed. Our review shows a significant amount of PFAS on turnout gear and their migration to untreated layers, and how turnout gear itself might be a potential source of PFAS exposure. PFAS from aqueous film-forming foams (AFFF), air, and dust of fire stations have been already established as potential exposure sources. Studies on firefighters’ cancer suggest that firefighters have a higher cancer risk compared to the general population. This review suggests that increased exposure to PFAS as an occupational hazard could be a potential cancer risk for firefighters.

## 1 Introduction

“Firefighter” and “cancer” are two words that have become unfortunately linked over the past decade. In 2022, the International Agency for Research on Cancer (IARC) re-classified the firefighting occupation as a “Group 1” carcinogen (carcinogenic to human) (Demers et al., 2022). This occupation was first classified in 2010 as “Group 2B” meaning possibly carcinogenic (IARC Working Group on the Evaluation of Carcinogenic Risks to Humans, 2010). Several studies examined the cancer risk and mortality rate among firefighters. Though studies have found inconsistent results, general indication of elevated risk of several cancers in firefighters have been reported, such as studies reported elevated summary risk estimates (SRE) for multiple cancers including non-Hodgkin lymphoma (NHL), myeloma, testicular and prostate cancers (LeMasters et al., 2006; Soteriades et al., 2019; Laroche and L’Espérance, 2021). Another study reported the cancer risk among firefighters with standardized incidence ratios (mSIR); this study found an increase in mSIR for skin melanoma and prostate cancer among firefighters (Casjens et al., 2020). Studies funded by the National Institute of Occupational Safety and Health (NIOSH) also reported elevated cancer mortality and incidence rates (Daniels et al., 2014; Daniels et al., 2015). Similarly, a study in Indiana found higher malignant cancer mortality for firefighters compared to non-firefighters (Muegge et al., 2018). Significantly elevated risk of skin, thyroid, testicular, and prostate cancer was also reported for male firefighters in Florida (Lee et al., 2020). Likewise, studies in many other countries and the United States also have found elevated rates of several types of cancer such as kidney, bladder, testicular, prostate, multiple myeloma, and non-Hodgkin’s lymphoma (Delahunt et al., 1995; Bates, 2007; Kang et al., 2008; Ahn et al., 2012). It has been documented that firefighting involves exposure to both known and potentially carcinogenic chemicals (Soteriades et al., 2019; Laroche and L’Espérance, 2021). Even though the exposure time could be shorter, levels of exposure could still be high (Jalilian et al., 2019).

Injuries caused due to the thermal and thermo-physiological comfort hazards during the firefighting are common among the firefighters (Mandal et al., 2021; Mazumder, 2021; Mazumder et al., 2022). In addition to thermal and thermo-physiological comfort hazards, exposure to harmful chemicals that may contribute to the increased cancer risk has become an increasing concern in the firefighting community (Trowbridge et al., 2020; Muensterman et al., 2021). Studies have shown that firefighters are exposed to hazardous substances during structural fires (Fent et al., 2014), overhaul phases (Jones et al., 2016; Oliveira et al., 2017) and vehicle fires (Fent and Evans, 2011; Jalilian et al., 2019). The compounds found in fire smoke and their toxicities vary considerably depending on the burning conditions and materials since every burn has its unique pattern (Golka and Weistenhöfer, 2008; Casjens et al., 2020). However, studies reported polycyclic aromatic hydrocarbons (PAHs) (Keir et al., 2020), 1,3- butadiene (Laitinen et al., 2012), metal (Keir et al., 2020), formaldehyde (Driscoll et al., 2016) and per- and polyfluoroalkyl substances (PFAS) (Jin et al., 2011; McGuire et al., 2014) are the most concerning chemicals (Muensterman et al., 2021). In addition, besides the fire scene, hazardous chemicals have also been found in fire station dust, aqueous film-forming foams (AFFF), contaminated fire equipment, and in turnout gear (Brown et al., 2014; Fent et al., 2015; Shen et al., 2015; Alexander and Baxter, 2016; Barzen-Hanson et al., 2017).

PFAS are a large class of fluorinated aliphatic chemicals, which have diversified use (Young et al., 2021). Due to their extreme toxicity, persistency, and bioaccumulation, these chemicals are a significant concern for the environment and human health (Blum et al., 2015; Cousins et al., 2016; Wang et al., 2017; Goldenman et al., 2019). PFAS are detected in the blood serum of over 98% of Americans (Calafat Antonia et al., 2007). The general population gets exposed to PFAS through drinking water, contaminated food, food packaging, cookware, indoor dust, and ambient air (Nadal and Domingo, 2014; Sjogren et al., 2016; Mastrantonio et al., 2018; Domingo and Nadal, 2019). Among many other PFAS, perfluorooctanoic acid (PFOA) and perfluorooctane sulfonate (PFOS) were most extensively used and studied. However, their use has been banned or heavily regulated in many countries for several years. Studies show that elevated levels of PFAS exposure is associated with adverse health effects such as testicular and kidney cancers (Vaughn et al., 2013; Vieira Verónica et al., 2013). Similarly, studies have also reported links between PFOA and cancers such as mesothelioma, prostate, testicular, and non-Hodgkin’s lymphoma (Vaughn et al., 2013; Chang et al., 2014). These four cancers are among the top eight that firefighters have increased risks of compared to the public (LeMasters et al., 2006; Daniels et al., 2014). Therefore, concern of PFAS exposure in the fire service and health risk of the firefighters are reasonable.

Increased risk of cancer in firefighters may be contributed from the occupational exposures to PFAS (Trowbridge et al., 2020). PFAS are particularly relevant to firefighting since these chemicals are used in firefighter protective clothing and equipment to impart water and oil repellency (Peaslee et al., 2020; Muensterman et al., 2021; van der Veen et al., 2022), as polymeric membranes in moisture barriers, in AFFF which are used in extinguishing fuel and oil fires (Kissa, 1994; Kissa, 2001; Barzen-Hanson et al., 2017), and recent studies have also found elevated PFAS levels in dust and air of fire stations (Henry et al., 2018; Young et al., 2021). Turnout gear worn by firefighters is extensively treated with fluoropolymers (one form of PFAS) or side-chain fluoropolymers to obtain the highest levels of water and oil repellency (Henry et al., 2018; Young et al., 2021). PFAS also includes polytetrafluoroethylene (PTFE), a highly repellent material used in the moisture barrier to limit the migration of water and bodily fluids through the turnout gear (Islam et al., 2020; Muensterman et al., 2021). Firefighters may also be exposed to PFAS chemicals during training and firefighting from fluorinated firefighting foams, which mainly contain PFAS from the sulfonated acids category (Dauchy et al., 2017). In addition, one recent study has found elevated levels of PFOS in the dust collected from the living area of fire stations: fifteen times higher than the median level (Young et al., 2021).

A few systemic review studies on human cancers and PFAS have been done (Sunderland et al., 2019; Imir et al., 2021; Steenland and Winquist, 2021; Boyd et al., 2022). Firefighters’ cancers (LeMasters et al., 2006; Ahn et al., 2012; Jalilian et al., 2019; Soteriades et al., 2019; Casjens et al., 2020; Lee et al., 2020; Laroche and L’Espérance, 2021) and PFAS in blood serum levels (Dobraca et al., 2015; Gribble et al., 2015; Rotander et al., 2015; Olsen et al., 2017; Graber et al., 2019; Kotlarz et al., 2020; Trowbridge et al., 2020) also have been studied extensively. The presence of PFAS in AFFF and hence contamination of groundwater has also thoroughly been studied (Rotander et al., 2015; Barzen-Hanson et al., 2017; Høisæter et al., 2019; Gonzalez et al., 2021). During the last decade, the presence of hazardous chemicals in fire station dust has also been reported by a few studies (Brown et al., 2014; Banks et al., 2020; Hall et al., 2020; Young et al., 2021). However, only a few recently published research studies mentioned the presence of PFAS in the turnout gear itself (Peaslee et al., 2020; Muensterman et al., 2021; van der Veen et al., 2022). Additionally, a recent study reviewed the PFAS exposure in firefighters from aqueous film forming foam (Rosenfeld et al., 2023). However, to the authors’ best knowledge, no comprehensive review on all the sources of PFAS that firefighters may be exposed to as an occupational hazard and their exposure pathways has been conducted. Therefore, this review covers explicitly the sources of PFAS as occupational exposure, their pathways, and how they might cause increased cancer risk in firefighters.

## 2 Methods

This review was performed in three steps. Firstly, introductory discussion was done on PFAS, which includes definition, classification, and chemical structures of most common PFAS. In the second step, the sources of PFAS exposure as firefighting occupational hazards were identified. The sources of PFAS including their chemistries and degradation pathways were discussed as identified in the literature. In the third step, elevated levels of PFAS in blood serum and associated increased rate of firefighters’ cancer were identified.

### Step 1: Classification of PFAS

The definition and classification of PFAS were discussed as mentioned in different literature. A universally accepted definition for PFAS is still missing. Commonly found PFAS were identified from the literature. In addition, PFAS that are mostly associated with fire service were also identified considering firefighters’ occupational hazard as reported in the literature.

### Step 2: Identification of sources of PFAS as firefighting occupational exposure

As reported in many peer-reviewed journals, one of the most common sources of firefighters’ exposure to PFAS is from AFFF, which is used to extinguish Class B fires. AFFF have been extensively used in firefighting and are one of the major causes of the ground water contamination with PFAS (Houtz et al., 2013; Cousins et al., 2016; Barzen-Hanson et al., 2017; Peaslee et al., 2020). Therefore, an occupational health concern of PFAS from AFFF has become more pervasive for firefighters (Laitinen et al., 2014; Gao et al., 2015; Grandjean and Clapp, 2015; Rotander et al., 2015; Mastrantonio et al., 2018). PFAS that could come out from the durable water- and oil-repellent (DWR) finish of the turnout gear have become an emerging topic very recently (Peaslee et al., 2020; Muensterman et al., 2021). PFAS have been detected in new and used turnout gear (Rewerts et al., 2018; Peaslee et al., 2020; Muensterman et al., 2021; Shinde and Ormond, 2021). The outer shell of the turnout gear is typically treated with side-chain fluorinated polymers, followed by a polytetrafluoroethylene-based (PTFE) moisture barrier (Holmquist et al., 2016; Henry et al., 2018). In addition, firefighters also could be exposed to PFAS while extinguishing structural or car fires, which may off-gas PFAS in the smoke due to the burning of different materials containing PFAS. Moreover, high concentrations of PFAS have been detected in the dust and air inside the fire stations (Hall et al., 2020; Young et al., 2021). Therefore, the surrounding environment of the fire scene and fire station is also a potential source of PFAS exposure (Hall et al., 2020; Muensterman et al., 2021; Young et al., 2021). Leaching of both volatile and non-volatile PFAS substances from the turnout gear and AFFF could be associated with the higher amount of PFAS in fire stations.

### Step 3: PFAS in blood serum and firefighters’ cancer

As discussed above, the firefighting occupation has been classified as a known carcinogen by IARC (Demers et al., 2022). Multiple pathways of PFAS exposure for firefighters are explored. Ingestion, inhalation, and dermal absorption are the most common exposure pathways as reported by literature. Elevated PFAS levels in blood serum and their association with certain types of cancer risk is discussed. Several studies on PFAS in blood serum (Gribble et al., 2015; Olsen et al., 2017; Graber et al., 2019; Kotlarz et al., 2020) and firefighters’ cancers have been reported (LeMasters et al., 2006; Soteriades et al., 2019; Casjens et al., 2020; Laroche and L’Espérance, 2021).

## 3 Definition and classification of PFAS

In 2011, PFAS was defined by Buck et al. (2011) as “aliphatic highly fluorinated substances that contain one or more C atoms on which fluorine atoms have substituted all the hydrogen atoms, so that the substance contains the perfluoroalkyl moiety C_n_F_2n+1_.” However, the Organization for Economic Co-operation and Development (OECD) reported in 2018 that molecules having fully fluorinated carbon atoms but lacking the -CF_3_ group, thus did not meet the requirements of the previous definition by Buck et al., 2011 (OECD, 2018). Therefore, to resolve this disagreement, OECD has proposed to define PFAS as: “fluorinated substances that contain at least one fully fluorinated methyl or methylene carbon atom (without any H/Cl/Br/I atom attached to it), e.g., with a few noted exceptions (represented by a carbon atom instead having H/Cl/Br/I atoms attached), any chemical with at least a perfluorinated methyl group (─CF_3_) or a perfluorinated methylene group (─CF_2_─) is a PFAS” (OECD, 2021). Therefore, it might be quite surprising but a universally accepted definition of PFAS is still wanted (Panieri et al., 2022).

**FIGURE 1:**
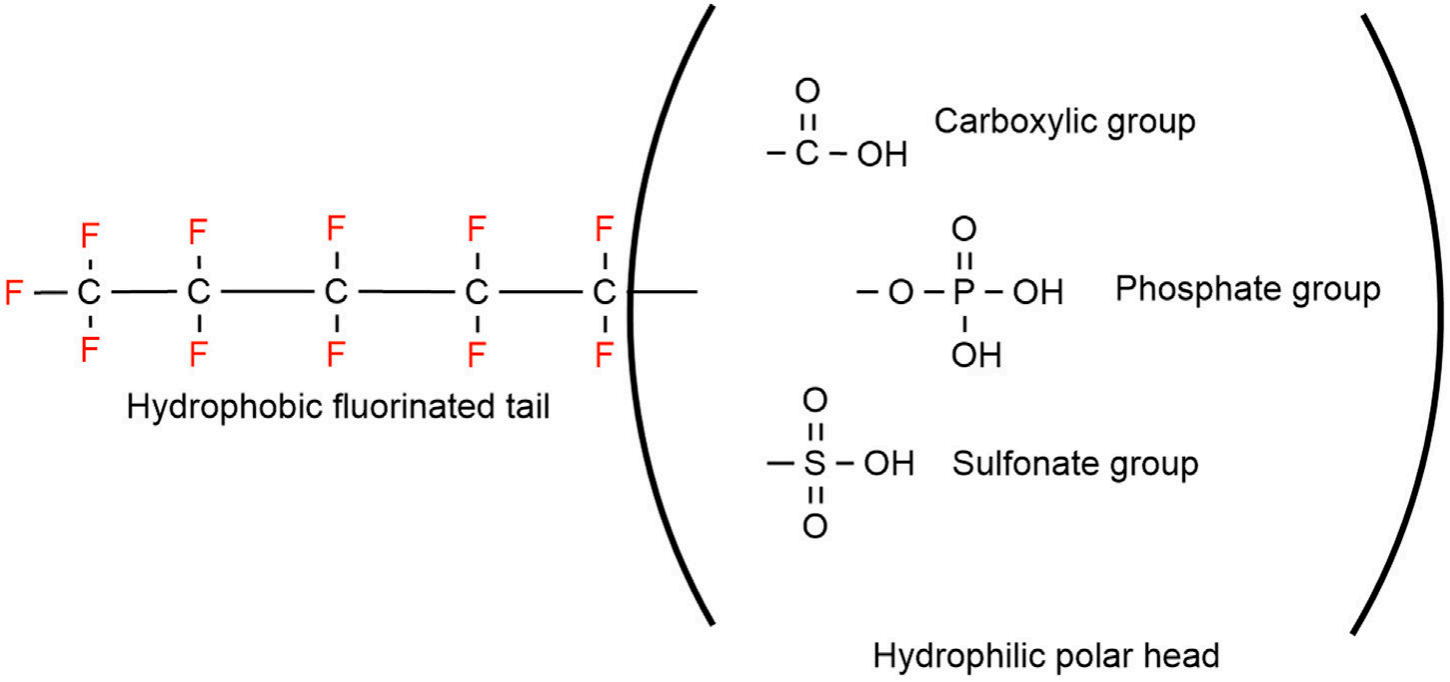
General structure of non-polymeric, perfluorinated PFAS substances.

Grouping the PFAS into two broad categories, polymeric and non-polymeric (Figure 1) molecules is the most adopted PFAS classification system, was proposed by Buck et al. (2011). The non-polymeric PFAS can be further divided into perfluoroalkyl and polyfluoroalkyl substances. The length of the fluorinated carbon chain is mostly used while classifying non-polymeric PFAS. The bioaccumulation, physico-chemical, and protein binding properties in addition to environmental fate distribution could be predicted by the length of the fluorinated carbon chain (Dai et al., 2013; Zhao et al., 2016; Johnson et al., 2021; Lu et al., 2021). If all the hydrogen atoms in each carbon atom have been replaced by fluorine except the terminal end the compound is known as a perfluoroalkyl substance (Figure 1) (Bank et al., 1994). The first available PFAS substances were perfluoroalkyl sulfonates (e.g., perfluorooctane sulfonate, C_8_F_15_SO_3^−^_, PFOS) and perfluoroalkyl carboxylic acids (e.g., perfluorooctanoic acid, C_7_F_15_COOH, PFOA), which were manufactured using an electrochemical fluorinated (ECF) process (Simons, 1949). High thermal and chemical stability and lowering surface tension in aqueous systems even at low concentration are unique properties for the widespread use of PFAS substances (Grajeck and Peterson, 1959; Holzapfel, 1966; Buck et al., 2011). Perfluoroalkyl acids (PFAAs) are the most commercially produced perfluorinated surfactants, which include perfluoroalkyl sulfonic acid (F(CF_2_)_n_SO_3_H, PFSA), perfluoroalkyl carboxylic acid (F(CF_2_)_n_CO_2_H, PFCA), perfluoroalkyl phosphonic acid (F(CF_2_)_n_P (=O)(OH)_2_, PFPA), and perfluoroalkyl phosphinic acid (F(CF_2_)_n_P (=O)(OH), PFPIA) (Buck et al., 2011).

**FIGURE 2:**
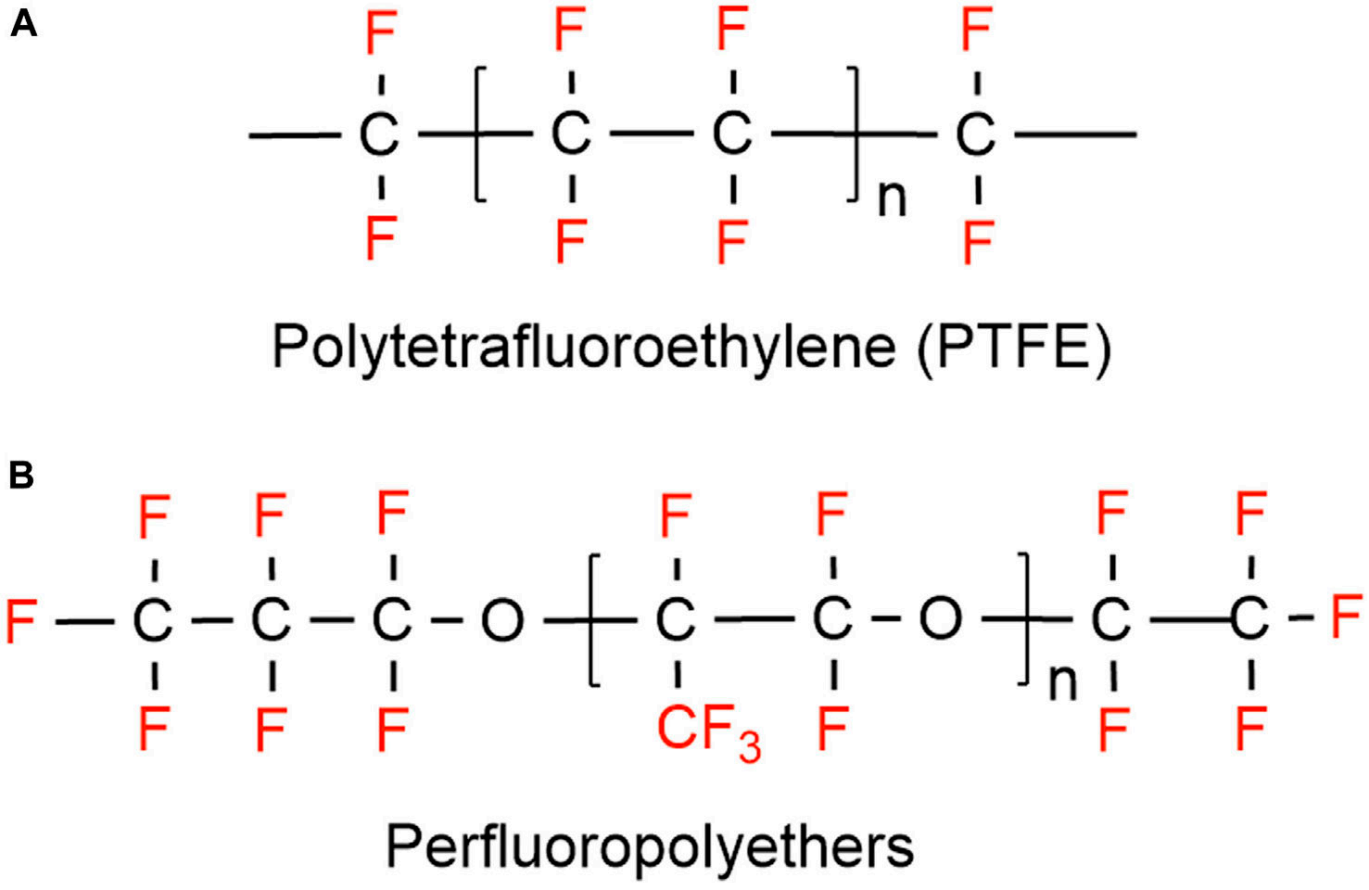
Polymeric PFAS substances, **(A)** PTFE, **(B)** perfluoropolyethers.

On the other hand, substances where not all the hydrogen atoms (but more than one) have been substituted with fluorine, are classified as polyfluoroalkyl substances (e.g., 6:2 fluorotelomer alcohol (FTOH)) (Smart, 1994). Different types of polyfluoroalkyl substances are: 1) fluoropolymers, substances where if not all, most of the hydrogen atoms of the carbon chain are replaced by fluoride atoms (e.g., PVDF, PTFE), 2) side-chain fluorinated polymers, where a poly/perfluorinated carbon chains are attached to non-fluorinated carbon chains (e.g., fluorinated acrylate polymers), 3) perfluoropolyethers where the backbone chain contains oxygens and fluoride atoms directly bound to the carbon chain, are classified as polymeric PFAS (Figure 2) (Panieri et al., 2022).

The classification of PFAS has been summarized in Table 1.

## 4 Sources of PFAS exposure

Firefighters could be exposed to several types of PFAS in the course of their daily duties. The common potential sources of firefighter occupational exposure to PFAS are discussed below.

### 4.1 Firefighter turnout ensemble

#### 4.1.1 Outer shell

**FIGURE 3:**
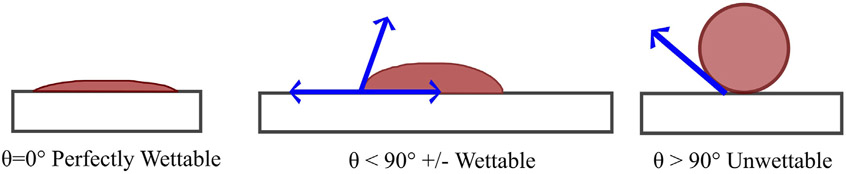
Wettability and contact angle of a substrate.

Water and oil repellent finishes provide the fabric with the ability to resist wetting when contacted by water and oily substances. With a high level of repellency, both water and oil drops should stay on the fabric surface and easily roll off (Sayed and Dabhi, 2014). A simple coating of paraffin or wax is the oldest form of water repellent finish, which would eventually wash out (Sayed and Dabhi, 2014). The fabric surface can be made hydrophobic by simply treating them with a hydrocarbon wax or silicone oil, that will repel water. However, finishing a fabric surface that will repel both water and oil is rather difficult (Mahltig, 2015). All oil repellents are also water repellents, but not all water repellents are oil repellents. The surface tension of most oils is below 15 dyn/cm, therefore, oil repellency required a surface tension below 15 dyn/cm, which is only achievable using fluorocarbon treatments. The repellency of water (surface tension 72 dyn/cm) is easily achievable with silicones, hydrocarbon waxes, and other technologies. The contact angle of water on the surface can be used to simply determine and classify the wetting behavior (Figure 3) (Marmur, 2006; Posner, 2012). Surfaces with 90° or higher contact angle against water are usually considered hydrophobic (Mahltig, 2015). Rough surfaces like textiles were reported to have even higher than 150° contact angle against water (Shibuichi et al., 1998). The term “surface tension” is used instead of contact angle to get an estimate of wetting. The surface tension is usually dependent on the chemical composition of that particular material (e.g., solid material or liquid). The surface tension of the textile surface needs to be lower than that of desired liquid (e.g., water, oil) to repel the liquid (Mahltig, 2015). Fluorinated compounds are a class of material that are repellent to both water and oil (Mahltig, 2015).

Fluorinated compounds exhibit excellent thermal and chemical properties, particularly important for durability against cleaning and care of the product such as laundering, drying, etc. In addition, the fluorinated compounds’ considerable reduction in surface tension property is essential to be classified as water- or oil-repellents (Sayed and Dabhi, 2014). The critical surface tension of textiles treated with acrylic polymers decreases rapidly with increasing chain length, which reaches to a minimum surface tension value at a nine-carbon chain (-(CH_2_)_8_-CF_3_) (Audenaert et al., 1999). As the surface tension significantly decreases the water and oil repellency increases considerably (Guo et al., 2008; Sayed and Dabhi, 2014; Gargoubi et al., 2020). Thus, a gradual increase in the fluorinated chain length from 1 to 9 enhances the water and oil repellency of the material (Sayed and Dabhi, 2014).

**FIGURE 4:**
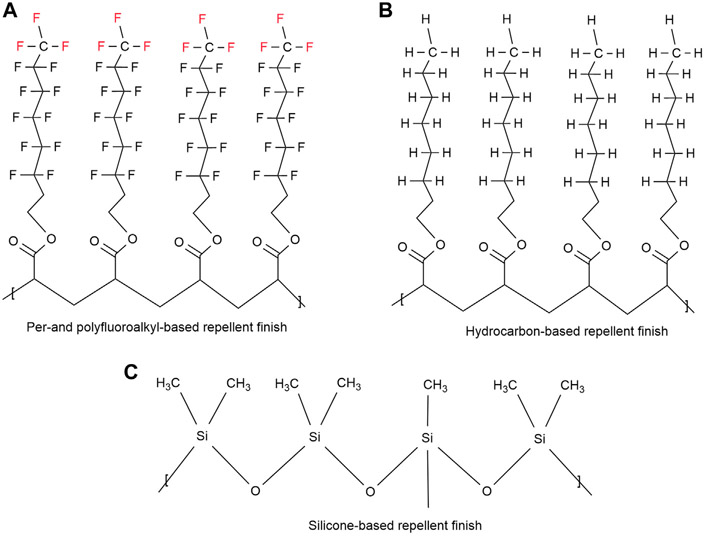
Structural examples of **(A)** side-chain fluorinated polymer, **(B)** hydrocarbon based repellent finish **(C)** silicone.

Most durable water repellents (DWR) typically have hydrophobic or oleophobic side-chains which are linked to a backbone polymer (Holmquist et al., 2016). These side-chains are based on silicones, hydrocarbons, or per- and polyfluoroalkyl moieties (Figure 4) (Dechant, 1985; Kissa, 2001; Holmquist et al., 2016). These repellent groups need to be closely packed and oriented to achieve the repellent property. The best water and oil repellency properties result from the fluorinated side-chain polymers since they have the lowest critical surface energy (Fox and Zisman, 1950). The silicone- and hydrocarbon-based side-chains provide excellent water repellency but lack in their ability to effectively repel most oils. Since all the DWR finishes function according to these principles, the more densely these groups are packed, the more hydrophobicity will result (Wang et al., 1997). Any conformation changes would cause fewer hydrophobic groups on the textile surface and hence less water repellency (Holmquist et al., 2016). In addition, a certain length of hydrophobic chain is required to protect the fabric from polar water droplets (Honda et al., 2005; Water, 2012).

Side-chain fluoropolymers are a form of PFAS used on the outer shell of firefighters’ turnout gear primarily to impart the durable water and oil repellency (Holmquist et al., 2016; Henry et al., 2018; Peaslee et al., 2020). Therefore, the outer shell material of turnout jackets and pants along with any other fabric surfaces in hoods, gloves, or boots that exhibit water and oil repellency have traditionally contained some form of fluorinated acrylate side-chain polymers, which may be built into the fabric or applied after the fabric is woven (Holmquist et al., 2016; Peaslee et al., 2020). Although the turnout gear has typically been treated with the side-chain fluoropolymer chemistries, many studies have reported the presence of non-polymeric PFAS on the textile materials (Gremmel et al., 2016; Schellenberger et al., 2018; Peaslee et al., 2020; Liagkouridis et al., 2021; Muensterman et al., 2021; van der Veen et al., 2022). A previous study reported that decomposing of side-chain polymeric PFAS could release non-polymeric PFAS (Washington et al., 2015). Fluorinated side-chain polymer treated durable water- and oil-repellents may release perfluoroalkyl carboxylic acids (PFCAs) and fluorotelomer alcohols (FTOHs) (Posner, 2012; Gremmel et al., 2016). Additionally, a study found two acylates; fluorotelomer acrylates (FTAC) in 28% and fluorotelomer methacrylate (FTMAC) in 58% of their air samples (*n* = 57) (Winkens et al., 2017). These volatile PFAS could potentially be the unreacted residual monomers from various side-chain fluorinated polymers (Russell et al., 2008). Therefore, degradation of the PFAS finish and textile surface due to ultra-violet light, washing, and high temperature exposures could release small volatile PFAS molecules as well as fabric dust/lint and subsequently serve as a source of PFAS exposure through inhalation, ingestion and dermal contact (Peaslee et al., 2020). For example, PFOA is a product of terminal degradation of C8-based side chain fluorinated polymer, which is a known persistent, bio-accumulative, and toxic substance. Similarly, alternative side-chains fluorinated polymers DWRs such as PFHxA (C6-based fluorotelomer) and PFBS (C4-based fluorotelomer) are equally persistent in the environment. The estimated elimination half-lives for selected perfluoroalkyl substances are provided in Table 2 (Substances and Registry, 2021).

Studies have reported significant quantities of PFAS in every layer of turnout gear including the thermal liner (Rewerts et al., 2018; Peaslee et al., 2020; Shinde and Ormond, 2021). Though most thermal liners are not intentionally treated with PFAS-based chemicals, studies found significant amounts of fluorine in all thermal liners tested (Peaslee et al., 2020; Muensterman et al., 2021). Some researchers have proposed that this finding suggests the migration of PFAS from the treated fabric layer to the untreated layer of clothing that may contact directly with the skin (Peaslee et al., 2020). Peaslee et al. (2020) found that most of the identified PFAS are short- and long-chain fluoroalkyl acids including PFOA. While that may be a possible route, another potential explanation is that PFAS in the smoke environment at a fire scene can easily infiltrate the turnout gear at the collar or waist interfaces as these ensembles are not vapor protective. A third possible route for the inner thermal liner to be contaminated with PFAS could be during the process of washing the gear. In this case the PFAS could be coming from the water source, a contaminated washer extractor, or from the contamination on the moisture barrier fabrics from the fire scene. In the washing process, the outer shell of the gear is separated from the moisture barrier and thermal liner and washed separately, so it is not as likely that any free PFAS from the outer shell would be able to transfer during laundering.

Muensterman et al. (2021) evaluated both the volatile and non-volatile PFAS in new turnout gear. The study found higher amounts of volatile PFAS than non-volatile PFAS in all layers of the turnout gear. The highest amount of both volatile and non-volatile PFAS was found in the moisture barrier, followed by the outer shell and thermal liner for volatile PFAS and thermal liner and outer shell for non-volatile PFAS (Muensterman et al., 2021). Longer chains fluorotelomer alcohols (FTOH) including 6:2, 8:2, 10:2, and 12:2 were measured in gear manufactured in 2003, suggesting that use of C8 chemistry at that time. However, shorter 6:2 FTOH were detected in the new turnout gear, which may reflect the switch from C8 to C6 chemistry in the early 2000s (2005–2015) (Muensterman et al., 2021). Studies suggest that significant amounts of PFAS may be released from the fluorinated textiles used in PPE for firefighters during the service of the garment (Peaslee et al., 2020; Muensterman et al., 2021). PFOA precursor material may leach from the side-chain fluorinated polymer, which could provide a route of exposure to the users of turnout gear (Peaslee et al., 2020).

Due to the undesirable toxicological and environmental behavior of long-chain side-chain fluorinated polymers, the industry is trying to move to alternative chemicals such as shorted chain fluorinated polymers and non-fluorinated durable water repellent finishes (Hill et al., 2017; Schellenberger et al., 2018; van der Veen et al., 2022). As a part of an alternative chemical analysis, Schellenberger et al. (2018) and Hill et al. (2017) evaluated the performance and durability of available fluorinated and non-fluorinated durable water repellent finishes on polyester and polyamide fabrics, which are usually used for outdoor performance clothing. van der Veen et al. (2022) evaluated the release of PFAS chemicals from C8 and C6-based fluorinated DWR on polyester and polyamide fabrics. As predicted, C8-based durable water repellent finishes showed the highest resistance to water and the highest contact angle, followed by C6 and C4-based DWR (Hill et al., 2017; Schellenberger et al., 2018). Hydrocarbon and silicone-based non-fluorinated DWR showed satisfying water repellency, but their repellency and durability performance were not consistent to make them alternative to fluorinated DWR (Hill et al., 2017; Schellenberger et al., 2018). Only the fluorine-based DWR showed resistance to oils, which was highest for C8-based DWR and reduced with the shorter chain length (Hill et al., 2017; Schellenberger et al., 2018). Similar patterns were observed in terms of durability and chain length of fluorinated DWR, decreasing water repellency was observed with decreasing chain length from 8 to 6 carbons. In addition, formation of longer chain perfluoroalkyl acids increased with ageing from C6 and C8-based DWR (van der Veen et al., 2022). However, non-fluorinated DWR showed good durability and was comparable in terms of durability and water repellency to the best fluorinated DWR (Schellenberger et al., 2018). C4-based DWR lost the oil repellency almost entirely whereas C8-based showed a strong drop after 10 cycles of washing (Schellenberger et al., 2018).

#### 4.1.2 Moisture barrier

In protective textiles, the moisture barrier is used to provide a breathable barrier that is resistant to water and many other liquids, however, it allows moisture vapor to pass through. In this way, the wearer gets protection from hot water and other toxic liquids while maintaining a heat balance by evaporating sweat vapors in high temperatures (Song and Lu, 2013; Shaid et al., 2018). The moisture barriers are either a hydrophobic membrane, coating, or microporous membrane (Holmes, 2000). Typically, a moisture barrier is composed of a two-layered membrane; a flame-resistant woven or non-woven fabric is bonded to a porous polymer film. The polymer film is typically composed of polyester, polyurethane, or expanded polytetrafluoroethylene (ePTFE). While all three polymers may perform similarly in moisture management, ePTFE exceeds the other two in terms of thermal protective performance and breathability. Polyester and polyurethane coatings start to degrade at around 150°C, and melt at 170°C–180°C. In contrast, the ePTFE membrane shows the high level of performance as the outer shell fabrics, which can withstand up to 350°C temperature. Therefore, most commercially available moisture barriers are ePTFE coated. In addition, moisture vapor transfer property of ePTFE membrane is the reason that these membranes are widely used compared to the polyester and polyurethane membranes. In the high heat environment, that added breathability is critical to limit the heat stress and reduce thermal burden.

As previously stated, PTFE is a form of PFAS that could be a potential source of PFAS exposure (Peaslee et al., 2020). The exposure could occur from the degradation of PFAS and be either inhaled or dermally penetrate (Peaslee et al., 2020). Studies have found that applied PFAS on textile materials degrade over time from heat, sunlight, and water exposure (van der Veen et al., 2020). A recent study found moisture barriers contain higher amounts of volatile and non-volatile PFAS compared to outer layers and thermal liners (Muensterman et al., 2021). The same study also reported total fluorine in all layers, which was measured by Particle-induced gamma ray emission (PIGE) and Instrumental neutron activation analysis (INAA) techniques. Both techniques gave the highest total fluorine value in moisture barrier compared to the outer layer and thermal layer (Muensterman et al., 2021). Though Peaslee et al. (2020) found PFAS in each layer of the turnout gear, they could not quantify the amount of fluorine on the moisture barrier since it was above the limit of detection in the (PIGE). Studies have reported off very high amount of fluorine concentration in the moisture barrier and were attributed to the PTFE fluoropolymer (Peaslee et al., 2020; Muensterman et al., 2021). Presence of PFOA in the newest moisture barrier (manufactured after 2012) had lowered PFOA than the minimum detection, which might be due to the shifting from long-chain PFAS solvent aids during the manufacturing of PTFE (Peaslee et al., 2020).

PTFE falls under the subgroup of fluoropolymer PFAS. Henry et al. (2018) suggested that fluoropolymers should be considered as low concern polymers, defined by OECD as polymers which have an insignificant effect on human health and the environment (OECD, 2009). However, the US Environmental Protection Agency (EPA) has now accepted side-chain fluorinated polymers as polymers of low concern considering the risk posed by polymeric PFAS but has not acted on fluoropolymers intrinsically (EPA, 2010). Lohmann et al. (2020) classified PFAS products as fluoropolymer substances, products, and finished articles. PTFE is an example of a substance where the chemical structure is known, whereas the commercial product is the actual product available in the market sold by different manufacturers, which may contain impurities from the production. The ePTFE moisture barrier used in firefighters’ turnout gear is an example of a finished article, which is manufactured from products (Lohmann et al., 2020). Though there is not enough evidence to justify keeping the fluoropolymers in the same toxicity group of non-polymeric PFAS, the emission of low molecular weight PFAS which are used as polymer processing aids during the manufacturing process of some type of fluoropolymers still can pose significant health and environmental effects (Henry et al., 2018; Hopkins et al., 2018; Brandsma et al., 2019; Lohmann et al., 2020). Therefore, Lohmann et al. (2020) suggested that fluoropolymer should not be considered as a polymer of low concern.

### 4.2 Aqueous film-forming foams

Fires involving hydrocarbon and other flammable liquids are Class B fires (Laitinen et al., 2014). Since water is more dense than liquid hydrocarbon fuels, it ends up at the bottom layer of a burning hydrocarbon surface and becomes ineffective at extinguishing the fire (Korzeniowski et al., 2018). In addition, the burning temperature of most fire scenes (≥175°C) is significantly higher than the boiling temperature of water (100°C), which causes vaporization of water to form steam. This could cause burn injuries and spread the fire rapidly (Korzeniowski et al., 2018). Therefore, aqueous film-forming foams (AFFF) which have excellent thermal stability and are capable of forming a film that sits on top of the fuel are used to extinguish this class of fires. Typical ingredients of aqueous film-forming foam are water, organic solvents, hydrocarbon surfactants, fluorosurfactants, polymers, and other additives (Peshoria et al., 2020). AFFF was developed in the 1960s and has been utilized to extinguish Class B fires ever since (Darwin et al., 1995). A considerable improvement in AFFF was achieved in 1970s through the manufacturing of fluorosurfactants-based foam. Per-fluorinated acids and salts of eight carbon atoms and other fluorinated compounds have been used mostly in film-forming foams (Kissa, 1994; Kissa, 2001). Naval Research Laboratory and 3M started working on AFFF containing fluorosurfactants-based on electrochemical fluorination (ECF) chemistry in the early 1960s, which led to the development of 3M’s “Lightwater” AFFF (Gipe and Peterson, 1972). ECF and the fluorotelomer are two chemistries used to synthesize fluorosurfactants. Perfluoroalkyl sulfonates (PFSAs) (e.g., perfluorooctane sulphonate [PFOS], C_8_F_17_SO_3^−^_) and perfluoroalkyl carboxylic acids (PFCAs) (e.g., perfluorooctanoic acid [PFOA], C_7_F_15_COOH) were the first commercially available fluorosurfactants, which were manufactured by the ECF process (Taylor, 1999; Kissa, 2001; Pabon and Corpart, 2002; Buck et al., 2012; Kempisty et al., 2016; Korzeniowski et al., 2018).

The strong carbon-fluorine bonds of the surfactants contribute to the high performance of AFFF (e.g., resistance to acid, alkali, oxidation, and reduction) even at high temperature. These surfactants play a unique role in reducing the surface tension of AFFF (Kissa, 1994; Kissa, 2001; Porter, 2013). “Surface active” properties of these fluorosurfactants come from the polar hydrophilic head and long non-polar fluorocarbon tail (Moody and Field, 2000; Buck et al., 2012; Baduel et al., 2017). The unique property of fluorosurfactants has made them almost irreplaceable in many unique industrial applications including extinguishing Class B fires (Jochyms et al., 2015). Firefighting foams are one of the reasons for the widespread presence of PFOS and PFOA, also known as long-chain fluorosurfactants, in the environment. Significant increase in using fluorosurfactants has caused an increased awareness concerning the adverse effect of AFFF on human health and the environment (Bursian et al., 2020). These chemicals are bio-accumulative in humans and wildlife, and persistent in the environment due to their strong carbon-fluorine bond. Discharges of these long-chain fluorosurfactants have been a concern by researchers globally (Lau et al., 2007; Post et al., 2012).

Non-biodegradable fluorosurfactants used in AFFF have a long life in the environment. There is a desire to find alternatives to fluorosurfactants due to their persistent nature (Wang et al., 2018). Usually, substances degrade or become immobilized when released into the environment but perfluorinated substances experience neither. Hence, these substances are highly soluble, transferable, and bioaccumulative (Peshoria et al., 2020). Bioaccumulation occurs when substances have affinity towards the biological component such as fat and protein and are stored in the fatty regions (Liu et al., 2011). Long-chain fluorosurfactants, which were used in traditional firefighting foam, have been recognized for their affinity toward liver, kidney, and blood protein (Pizzurro et al., 2019). Fluorosurfactants have been identified as the principal component of AFFF that causes their negative environmental impacts (Høisæter et al., 2019).

Different PFAS such as perfluorooctanoic acid or its salt, perfluorooctanesulfonic acid or its salt, and perfluorohexanesulfonic acid have been detected in blood, human serum, and milk (Calafat Antonia et al., 2007; von Ehrenstein et al., 2009; Gützkow et al., 2012). Studies on perfluoroalkyl acids (PFAAs), including PFOS and PFOA, have shown that these compounds may affect total and LDL cholesterol and are associate with breast cancer (Steenland et al., 2009; Nelson et al., 2010; Bonefeld-Jorgensen et al., 2011). A study conducted by Shaw et al. (2013) found that firefighter exposure to fire retardant chemicals, such as polychlorinated and polybrominated dibenzo-p-dioxins and dibenzofurans have similar effects.

Elimination of long-chain fluorosurfactants, while continuing to deliver the unique and valuable properties of fluorosurfactants, led manufacturers to develop short-chain alternatives (Peshoria et al., 2020). Studies have shown that short-chain alternatives have a less impact on environment and human health compared to long-chain chemistry (EI Corporation, 2014; Buck, 2015). However, there is still controversy about whether short-chain PFAS have less impact on environment and human health compared to long-chain PFAS. Some sources say that short chain PFAS are better while some do not. Carbon chain lengths greater than or equal to six within the perfluoroalkane sulfonate (C_n_F_2n+1_SO_3_(H)) (PFSA) family are considered as long-chains. However, in the perfluorocarboxylic acid (C_x–1_F_2x–1_COOH) (PFCA) family carbon chain lengths greater than or equal to eight are considered as long-chains (Kempisty et al., 2016; Korzeniowski et al., 2018). This is due to the significant differences in toxicity and bio-accumulation properties between the two families (Gannon et al., 2011).

A study found increased levels of perfluorohexanoic acid (PFHxA) and perfluorooctanoic acid (PFNA) in the firefighters’ blood serum after the training session, where the firefighters were exposed to AFFF (Laitinen et al., 2014). However, these elevated PFAS were not the main components of the AFFF. It was hypothesized that long-chain fluorotelomers decomposed during the jet fuel fire (Laitinen et al., 2014). Firefighters’ exposure to PFAS from AFFF could be through inhalation and dermal routes. Ingestion is possible from hand-to-mouth transfer from contaminated turnout gear after a suppression or training (Laitinen et al., 2014).

### 4.3 Fire scene

**FIGURE 5:**
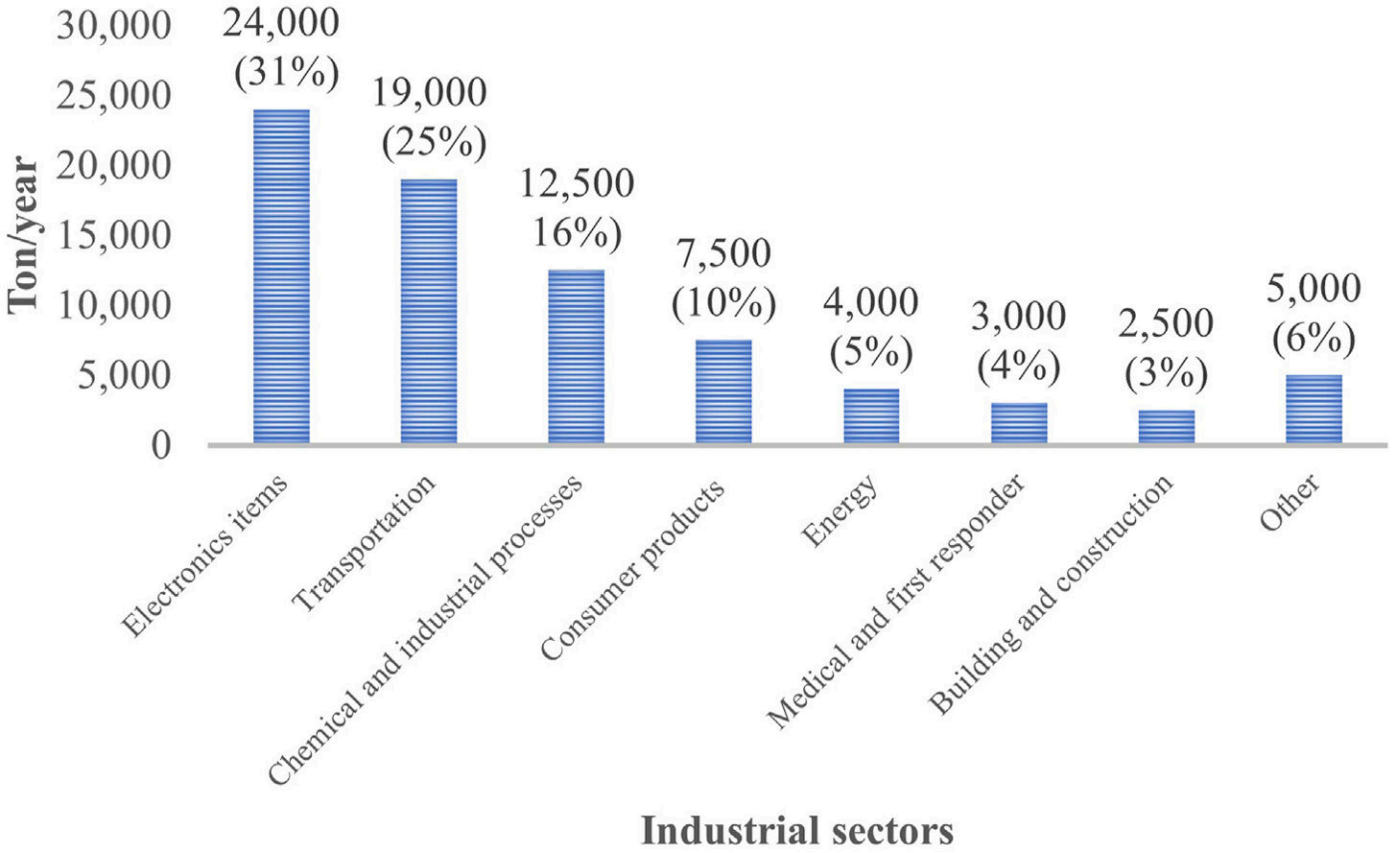
Use of fluoropolymers (in tons) by United States industrial sectors in 2018 (Kempisty and Racz, 2021).

Research found that the risk of PFAS exposure is higher for firefighters compared to the general public (Laitinen et al., 2014; Grandjean and Clapp, 2015; Rotander et al., 2015; Mastrantonio et al., 2018; Peaslee et al., 2020). Although the use of PFOA and PFOS has been stopped mostly over the last 15 years, these compounds were commonly used in furniture, carpet, paper, or industrial products (Beecher and Brown, 2018; Mazumder and Islam, 2021). Many of the products containing these compounds are still available in our daily life. PFAS are also currently used in several applications including apparel, semiconductors, and pharmaceuticals (Figure 5) (Rizzuto, 2020). The highest quantity of PFAS in the USA is used in electronics. PFAS are used in electronic products such as wire, cables, liquid crystal or flat panel displays. Electronic devices used as testing equipment like sensors and fluids used for heat transfer are also required to use PFAS as fluorinated compounds significantly improve the applications of these devices (Glüge et al., 2020; Tansel, 2022).

PFOA and PFOS can be produced from these PFAS containing products once these precursor compounds break down. During a fire, PFAS compounds (both polymeric and non-polymeric) may break down into precursor compounds like fluorotelomers alcohols that can further degrade into the terminal PFOA, PFOS, or other fluorinated compounds and be released into the environment. Firefighters or first responders on the fire scene would be exposed to these compounds during firefighting activities in emergency response and during training scenarios.

Tao et al. (2008) investigated perfluorochemicals from blood plasma samples of first responders due to the exposure to dust and smoke generated from the collapse of the World Trade Center (WTC). Plasma levels of PFOA and perfluorohexane sulfonate (PFHxS) were found more than twofold in the body of first responders compared to the general public (Tao et al., 2008). Firefighting foam, furniture, or other materials used inside the buildings might be the possible source of perfluorochemicals like PFOS and PFOA which were released in and around the site followed by the collapse. The findings of this research suggested that greater exposure to smoke and dust might cause the high concentrations of fluorochemicals in the body of first responders. They also found that smoke exposure contributed more compared to dust exposure to elevate the concentration of PFOA and PFHxS. Like the WTC incident, firefighters perform their duties in numerous structural burns and expose themselves to a high concentration of toxic chemicals including fluorochemicals present in smoke and dust generated from the burn of pulverized building materials and contents.

Previous studies showed that various volatile and semi-volatile compounds can off-gas from the surface of turnout gear for a certain period which raises concern regarding PFAS as firefighters get exposed to AFFF on a fire scene or smoke generated from PFAS-containing consumer products (Fent et al., 2015; Fent et al., 2017; Mayer et al., 2019). If generated PFAS from the fire scene can adsorb to the surface of ensembles, the exposure will be transferred to where the gear is stored in the fire station. Therefore, further research is required to investigate whether PFAS-contaminated gear works as an additional source of PFAS presence inside the fire station.

### 4.4 Dust and indoor air

According to the US EPA, adults and children ingest almost 30 mg and 60–100 mg of indoor dust per day respectively (Moya et al., 2011). For the general public, indoor environments, including dust and air, are considered as a source of PFAS or organic fluorine exposure (De Silva et al., 2012). PFAS concentrations for compounds such as PFCAs, PFSAs, FTOHs, and perfluorooctylsulfonamides (PFSAm) have been reported to be higher in indoor air compared to outdoor air (Shoeib et al., 2005; Harrad et al., 2010). Shoeib et al. (2005) found that a semi-volatile neutral precursor, PFSAm, was 10–20 times higher in indoor air compared to outdoor air. A similar trend was observed for some natural volatile precursors such as PFSAs and PFCAs when indoor and outdoor air were compared (Shoeib et al., 2011). Fraser et al. (2012) investigated indoor air from 30 offices and found significantly high concentrations of polyfluorinated compounds (PFC) inside these offices. They also observed the highest FTOH in the most recently constructed building among other offices and concluded that off-gassing of FTOHs from the new carpets or furniture from the new buildings is mostly responsible for this elevated concentration (Fraser et al., 2012).

In addition to turnout gear, firefighters also store other sources of PFAS, such as AFFF and upholstered furniture, in fire stations. With these items, firefighters may bring residual PFAS contamination into the fire station which can act as a source of PFAS by contaminating indoor air or releasing air-prone dust. Previously, dust has been found to be an important exposure pathway of flame-retardant chemicals for firefighters (Jones-Otazo et al., 2005; D’Hollander et al., 2010; Stapleton et al., 2012; Mazumder and Islam, 2021). Therefore, it was required to investigate the role of indoor air and dust as the potential source of PFAS exposure for firefighters. Recent research found significantly high concentrations of PFAS in fire station dust which is alarming for firefighters as they spend most of their shift inside fire stations (Hall et al., 2020; Young et al., 2021). Young et al. (2021) analyzed dust samples from 15 fire stations in Massachusetts targeting 24 PFAS. The median dust concentration of these 24 PFAS in fire stations was 98.7 ng/g with N-EtFOSAA, 6:2 FtS, PFDS, 8:2 FtS, and PFOS being the prominent components. The targeted 24 PFAS account for only 1.2% of the total detected fluorine mass which indicates the presence of unidentified non-polymeric and polymeric PFAS in the dust (Robel et al., 2017; Schultes et al., 2019). They also found a higher concentration of fluorine from the dust of turnout gear locker rooms compared to the dust collected from living rooms in fire stations. Since turnout gear is kept in locker rooms in some stations (in others it is kept in the engine bay), this demonstrated that turnout gear could be a major source of PFAS exposure to firefighters.

PFAS contaminated dust could be originated from external contamination during firefighting activities or the PFAS intentionally added to gear. Laundering also may release some of the PFAS used in turnout gear because it has been found that side-chain fluoropolymers from the individual fiber can be released during washing of outdoor jackets (Schellenberger et al., 2019). When the fluorine-containing fibers release in the environment, the backbone of the fluorinated polymer may be cleaved over time and form short-chain perfluoroalkyl acids. Little information has been obtained yet regarding the release of PFAS compounds used in firefighting ensembles due to laundering. Therefore, the contribution of laundering to the release of PFAS compounds should be explored further. Hall et al. (2020) investigated indoor dust samples collected from 49 fire stations located both in United States and Canada and found 6:2 FTOH to be the most prominent PFAS detected (760 ng/g dust). They also collected dust from North Carolina homes and found significantly higher PFOS, PFOA, PFHxS, PFNA, and 6:2 diPAP in fire station dust compared to residential dust. Median dust levels of PFOS, PFHxS, and 6:2 diPAP were 15 times, 3 times, and 2.5 times higher, respectively, in fire station dust compared to home dust (Hall et al., 2020). Although this finding does not guarantee similar trends all over the country, this indicates higher exposure to PFAS inside fire stations compared to residential areas. Authors found that PFAS concentration in the dust of United States fire stations is significantly higher compared to the dust collected from Canadian fire stations although they hypothesized that differences in data collection period could be responsible for this difference (Hall et al., 2020). PFOS was predominantly present in the dust collected from United States fire stations.

PFAS have been detected on almost every layer of turnout gear since PFAS are used in the turnout gear to impart heat stable and oil resistance properties (Peaslee et al., 2020). Young et al. (2021) found PFAS on the surface of turnout gear collected by gear wipes. The highest detected amount of PFAS was 84,500 ng/wipe whereas over 50% of the total PFAS mass on gear wipes consisted of perfluoroalkyl carboxylic acids (PFCAs). PFOA, PFHxA, PFDA, PFNA, PFHpA, and 8:2 FtS were the most frequently detected PFCAs. The concentration of volatile PFAS is found higher in turnout gear compared to non-volatile PFAS (Muensterman et al., 2021). PFAS used in gear can degrade over time from exposures to heat, water, and sunlight, which indicates used PFAS in firefighters’ ensembles also can be shed into the environment and act as a source of exposure for firefighters (Rankin et al., 2014; Rankin, 2015; van der Veen et al., 2020). Peaslee et al. (2020) compared between 10-year-old unused ensembles and ensembles used for 10 years of service. They found that used samples lost 80% of total fluorine from the outer shell surface within 10 years of service. The loss of fluorine indicates that PFAS likely shed off into the external environment. They also ran methanolic extraction of the dust samples collected from the workstation floor of a PPE processing facility and observed n-Et-FOSAA. Dust sample analysis indicates that it likely originated from PPE ensembles rather than AFFF since short and long fluorotelomer sulfonate and fluoroalkyl sulfonates were not detected in the samples (Place and Field, 2012; Barzen-Hanson et al., 2017; Peaslee et al., 2020). Methacrylate esters are generated from Et-FOSE which acts as the backbone polymer of fabric finishes and Et-FOSE is eventually turned into Et-FOSAA when it is decomposed and hydrolyzed (Plumlee et al., 2009; Benskin et al., 2013; Liu and Avendano, 2013; Wang et al., 2014; Washington and Jenkins, 2015). Therefore, detection of Et-FOSAA in dust samples indicates side-chains of fluoropolymers are already degraded. Since Et-FOSE was presumably oxidized into PFOA, decay products from Et-FOSAA may eventually expose firefighters to PFOA precursor materials and enter into the firefighters’ body (Plumlee et al., 2009).

## 5 Uptake pathways

The three primary routes of exposure are ingestion, inhalation, and dermal absorption. PFAS is known to be distributed throughout the environment, so all three routes are worth considering (Fraser et al., 2012; Peaslee et al., 2020). Ingestion of food and drink has been identified as the prominent uptake pathway of PFAS to the general public (Poothong et al., 2020). For instance, AFFFs and other environmental contaminants disperse through groundwater and soil from nearby sites (Backe et al., 2013; Houtz et al., 2013; Dauchy et al., 2019). PFAS can then accumulate in the human body directly from contaminated water or through foods including red meats, eggs, vegetables, snacks, seafood, animal fat, etc. (Huang et al., 2020).

Since dietary consumption is a concern for the general public, firefighters should also be concerned (Trudel et al., 2008; Fromme et al., 2009; Haug et al., 2011a; Egeghy and Lorber, 2011). One study identified produce grown at a fire station as a major source of PFAS exposure (Tefera et al., 2022). This study showed that the consumption of eggs produced at firehouses appeared to be the leading route of PFAS exposure, followed by the consumption of fruits and vegetables and skin contact with dust-contaminated surfaces. Based on median and typical exposures, food consumption accounted for 82% of the total PFAS intake of firefighters, followed by incidental ingestion and dermal exposure to PFAS in dust (15%). Accidental ingestion and skin absorbed PFAS from soil and utensil cleaning resulted in <1% (Tefera et al., 2022). These findings broadly support the work of many other studies in which food consumption was identified as the most important route of PFAS exposure (Haug et al., 2011a; Haug et al., 2011b). Estimated dietary intake of PFAS in this study was much higher than previous estimates of dietary intake for the general population (Chain et al., 2018). Although this study focused on occupational firefighters, the dietary exposure routes identified here have wider relevance to the general public, especially those who consume food grown in or near PFAS-contaminated areas. This study is the first to describe a unique dietary PFAS exposure pathway in the context of professional firefighters.

In addition, due to occupational activities, the risk of dermal and respiratory absorption of PFAS is significantly higher for firefighters. For instance, PFAS used in turnout gear may transfer from the gear to the firefighter’s skin. Another firefighting specific source is contact with AFFFs. Besides that, smoke generated from consumer products containing PFAS can contaminate turnout gear and skin as well as being an inhalation hazard. While no studies confirm the sources of PFAS on the fire scene, PFAS is still commercially used, so it should be present in soot or smoke. There is a need to determine the disposition and amounts of PFAS generated and released during fire suppression activities.

From these sources, the inhalation pathway should be limited assuming the proper and consistent use of self-contained breathing apparatuses (SCBA). Any volatilization of contaminants from smoke, AFFF, or the gear itself should not be inhaled during use of SCBAs during active fire supression. However, the use of SCBAs for each firefighter on a fire scene throughout the entire duration of the incident is not consistent across or within departments. Incident commanders and pump operators rarely use SCBAs on scene, and firefighters on interior or exterior attack may remove their facepieces during or after on-scene decontamination when they are still within reach of the smoke. This common misconception has resulted in few studies investigating the contribution to PFAS exposure of inhalation at a fire scene. Aside from the inhalation route, dermal absorption is most likely the next primary uptake pathway. Previous research has reported that chemical contaminants can transfer from fabrics to skin (Blum et al., 1978; Appel et al., 2008; Rossbach et al., 2014). Research needs to be done to determine the extent that PFAS can off-gas or leach from fabrics and then transfer and absorb into skin.

PFOA is a fluorochemical that has been detected in the blood of most Americans during the last decade, though the concentration is starting to decrease. Franko et al. (2012) investigated the possibility of dermal penetration of PFOA into human skin. They found that PFOA can penetrate human skin and dermal absorption of fluorochemicals could be a major route of PFAS exposure (Franko et al., 2012). The study showed that PFOA at pH 2.25 has a 3-order of magnitude increase in the skin permeability coefficient compared to a pH of 5. This difference in pH affects the ionization state of PFOA, which has a pKa of 3.8 (Franko et al., 2012). Below the pKa value, PFOA is mostly un-ionized. Un-ionized compounds have better lipid solubility than their ionized counterparts and can more readily penetrate the lipid matrix of skin. The pH of skin is approximately 5.5 so PFOA is expected to be mostly ionized in skin. Although in general, PFOA penetration should be low for human skin as most of the exposures occur in the ionized state (Table 3), it is still a concern in the case of a higher level of exposure.

*In vivo* methods have been used to analyze the dermal absorption and effect of PFAS. Abraham and Monien (2022), was concerned about the absorption of PFAS through cosmetics so they measured the amount of dermally absorbed PFOA from a prepared sunscreen solution. The sunscreen solution (^13^C4-PFOA concentration of 3.7 μg/g) was directly applied to the individual’s whole body surface and allowed to maintain on the skin for 48 h before the individual took a shower. Blood samples were taken, and plasma analyzed with UHPLC-MS/MS. Starting at the initial application of sunscreen, PFOA was detected to be increasing for the first 10 days then stayed at a level rate for the next 110 days of sampling. Researchers estimated that 1.6% of the applied dose of the PFOA was absorbed over the exposure. This study helps illustrate that in this cosmetic dosing vehicle, PFOA can be absorbed dermally and is not quickly metabolized or excreted. A study showed that brominated and chlorinated chemicals, which are structurally related to PFAS, are dermally bioavailable and can result in significant body burdens. This suggests that dermal exposure could be an important exposure pathway to PFAS, especially with the consumer products that are relevant to dermal contact (i.e., water-proof fabrics, cosmetics) (Ragnarsdóttir et al., 2022).

Other *in vivo* studies have focused more on the toxicological impacts of PFAS. Franko et al. (2012) looked at cytokine expression on mouse skin and found no significant expression compared to the control. Dermal exposures of PFBA and PFOA have been found to increase liver weight and alter the PPAR pathway in mice (Shane et al., 2020; Weatherly et al., 2021). Han et al. (2020) also investigated the effect of dermal exposure to several PFAS (PFOA, PFHpA, PFHxA and PFPeA) on human skin and found no significant toxicological effects. The *in vivo* study of PFHpA found tubular and hepatocellular necrosis and germ cell degradation. While knowing the potential impacts is important, a large dose was used for the *in vivo* study and seems unreasonable for human exposure. Fasano et al. (2005) used rat and human skin to investigate PFOA penetration if dermal contact occurs. They found that PFOA can penetrate through both human and rat skin although the penetration rate is 34 times slower in human skin compared to rat skin. There is a need for more data on the dermal toxicological effects of PFAS, the mechanisms of action, and how it relates to humans. Moreover, no research has been conducted particularly addressing the dermal absorption of PFAS from new or contaminated turnout gear to firefighters.

One study conducted on children found that dust ingestion may have similar impacts to dietary ingestion of PFCs (Egeghy and Lorber, 2011). PFAS has already been demonstrated to be found in large concentrations inside fire stations (Hall et al., 2020; Young et al., 2021). Therefore, ingestion of dust and indoor air inside fire stations are major sources of PFAS for firefighters through the respiratory tract besides most commonly recognized dietary sources like food and water (Sunderland et al., 2019; Chain et al., 2020). PFAS exposure to firefighters may also impact their children. In addition to potentially carrying PFAS into their homes, it can be transferred through breastmilk. One researcher found a strong correlation between higher PFAS concentration in maternal serum and breast milk (Inoue et al., 2004). Women make up approximately 5.1% of the firefighting population, their exposures need to be considered (Hulett et al., 2007; Trowbridge et al., 2020). This indicates children of female firefighters may get exposed to PFAS for a long-term at an early age through placenta or lactation (Inoue et al., 2004; Gützkow et al., 2012; Cariou et al., 2015; Chen et al., 2017). This could make children more vulnerable to adverse health effects (Inoue et al., 2004).

Young et al. (2021) recommended that as firefighters spend almost 72% of their time on a 24-hour shift inside the fire station, there needs to be minimal PFAS in the station. Indoor air samples need to be monitored regularly to determine the PFAS exposure level. To minimize both dermal and respiratory exposure to PFAS particles, firefighters should use protective ensembles including SCBA and turnout gear consistently at the fire scene. Wearing PFAS free clothing under protective ensembles, storing PFAS containing ensembles from other clothes, and washing hands after touching ensembles are also necessary to reduce the risk of dermal or respiratory exposure to PFAS (Young et al., 2021).

## 6 PFAS in blood serum

Historically, firefighters have been exposed to harmful chemicals from fire smoke and firefighting foams containing high levels of various PAHs and PFAS (Jalilian et al., 2019; Gasiorowski et al., 2022). Additionally, a new concern has been raised regarding firefighters’ exposure to PFAS through turnout gear. Several studies have observed the association between PFAS exposure and a range of adverse health outcomes (Graber et al., 2021). Although PFAS are potentially harmful to human health, the exact threshold at which these risks may increase has remained unknown (Gasiorowski et al., 2022). Like other chemical substances, their ability to produce adverse health effects depends on exposure circumstances, such as magnitude, duration, and route of exposure (Fenton et al., 2021). In addition, individuals’ age, sex, ethnicity, health status, and genetic predisposition may also influence adverse health outcomes (Fenton et al., 2021). Nonetheless, several long-chain PFAS have been associated with cancer risks (Temkin et al., 2020). Among them, PFOA has been classified as a possible human carcinogen for kidney and testicular cancers by the International Agency for Research on Cancer (Rotander et al., 2015; IW Group, 2016).

Per- and polyfluoroalkyl substances are particularly concerning because of their persistent, bioaccumulative properties (Li et al., 2018; Graber et al., 2021). They can stay in the human body for long periods of time without being changed and can interfere with the bodily functions (Li et al., 2018). They accumulate in organisms by binding to plasma protein and sequestration into the liver, kidney, and lungs (Meegoda et al., 2020). The ability to bind to blood proteins, slow urinary excretion, and low clearance are predictors of a bioaccumulative chemical with a long half-life (Tonnelier et al., 2012). Long-chain PFAS such as PFOS and PFOA have a half-life of 5.4, and 3.8 years, respectively (Li et al., 2018). PFOS alternatives, such as perfluorohexanesulfonic acid (PFHxS) has a much longer half-life of 8.5 years (Li et al., 2018). Other short chain alternatives, such as perfluoropentanoic acid (PFPeA) and perfluorobutane sulfonate (PFBS), have a shorter half-life of a couple of weeks (Li et al., 2018). Although PFOA and PFOS have been extensively studied, the health outcomes of their alternatives have not been studied as thoroughly. The presence of these PFAS alternatives in the human body is still a matter of concern despite having a shorter half-life and being at a low level.

Due to their widespread use and ubiquitous presence in the environment, most Americans have background exposure to some PFAS (Graber et al., 2021). However, firefighters’ exposures can be occupationally related as they are exposed to PFAS through multiple pathways, making them more vulnerable to exposure. The commonly detected PFAS among firefighters are PFOA (ranging from 1.15 to 2.15 ng/mL), PFOS (ranging from 4.11 to 8.63 ng/mL), PFHxS (ranging from 1.83 to 6.15 ng/mL), PFNA (ranging from 0.46 to 0.97 ng/mL), PFDA (ranging from 0.25 to 0.31 ng/mL), and PFUnDA (ranging from 0.11 to 0.18 ng/mL) (Trowbridge et al., 2020; Graber et al., 2021). Firefighters with a history of using AFFF have elevated serum levels of PFOS and PFHxS (Rotander et al., 2015). In this regard, several studies reported higher serum levels of some long-chain PFAS among firefighters compared to the general population of similar demographic subsets (Dobraca et al., 2015; Rotander et al., 2015; Leary et al., 2020; Trowbridge et al., 2020; Graber et al., 2021) (Table 4). These studies used participants from the National Health and Nutrition Examination Survey (NHANES) as the representative of the US general population. The survey is administered by the Centers for Disease Control and Prevention (CDC) and published on a 2-year cycle.

One recent study conducted among male New Jersey volunteer firefighters observed that average serum concentrations of PFNA (+53%), PFDA (+39%), and PFDoA (+50%) were significantly higher than the NHANES population (Graber et al., 2021). Another study in the Southwest Ohio region found higher serum PFOS (+29%) and PFHxS (+74%) concentrations among suburban firefighters than the US adult male (NHANES 2015 to 2016 data) (Leary et al., 2020). In contrast, the serum levels of PFOS (−43%) and MeFOSAA (−88%) were significantly lower than the general population (Graber et al., 2021). The lower serum level coincides with the phase-out of some long-chain PFAS, including PFOA and PFOS, from consumer products and firefighting equipment (Rotander et al., 2015). However, these compounds are anticipated to persist for many years because of their long half-lives. The study also observed a positive association between serum levels of PFDA and PFDoA with years of firefighting (Graber et al., 2021). The findings were consistent with a 2015 biomonitoring study of Southern California firefighters (Dobraca et al., 2015). Both studies reported that the average serum concentrations of perfluorodecanoic acid (PFDA) were three times higher than those in NHANES participants. An all-female cohort study conducted in San Francisco found similar results, where women firefighters had higher geometric mean concentrations of PFNA, PFHxS, and PFUnDA than the office workers (Trowbridge et al., 2020). This study was unique in the sense that it compared firefighters to other non-firefighters in the same geographic area. One thing to be noted is that comparing with NHANES samples does not necessarily reflect what is the overall PFAS exposure scenario in a particular area.

Though these study findings were consistent across different US geographic locations regarding higher serum levels in firefighters’ blood, serum profile and levels of PFAS varied across different areas. One study in Southern California did not find any detectable serum levels of PFDoA (Dobraca et al., 2015), while another study in New Jersey detected significantly elevated serum levels of PFDoA (Graber et al., 2021). The PFDoA serum level was twice as high as in the NHANES participants (Graber et al., 2021). Similarly, the mean serum levels of PFHxS and PFOS varied across location. Background exposure stemming from consumer product use (Lindstrom et al., 2011), food and drinking water contamination (Xu et al., 2021), proximity to industrial sites (Steenland et al., 2009; Schroeder et al., 2021), and military airbases (Xu et al., 2021) may also contribute to the high levels of some PFAS in different geographical areas. In a study, female firefighters assigned to the airport fire station reported having two times higher PFNA levels than firefighters assigned to other stations in San Francisco (Trowbridge et al., 2020). Airport firefighters in the Southwest Ohio region had 21%–62% higher PFAS serum concentrations than suburban firefighters (Leary et al., 2020). Likewise, a study in Finland observed that training activities involving AFFF to extinguish jet fuel fires increased firefighters’ serum PFNA concentrations (Laitinen et al., 2014). Although PFNA is not considered a main ingredient in AFFF, these observations indicate other possible PFAS sources in fire scenes than firefighting foam. Observational studies have found female firefighters had lower levels of most PFAS compared to male firefighters (Wong et al., 2014; Trowbridge et al., 2020). Perfluorinated compounds have a higher affinity toward fatty acid-binding proteins in the blood, and therefore some PFAS might be eliminated from the body during blood donation or menstruation cycles (Jones et al., 2003; Rickard et al., 2022). Blood donor firefighters were found to have lower PFAS levels than non-donor firefighters with equivalent exposure (Rotander et al., 2015), suggesting plasma donation could be a possible elimination pathway (Wong et al., 2014; Silver et al., 2021).

## 7 PFAS exposure and cancer

There has been extensive research examining possible relationships between PFAS levels in blood and harmful health effects in people (Fletcher et al., 2013; Wielsøe et al., 2015; Croce et al., 2019; Jain and Ducatman, 2019; Wang et al., 2019; Li et al., 2021). PFOA and PFOS are two of the most widely studied PFAS compounds, followed by PFHxS and PFNA (Kim et al., 2021). These studies suggest that high levels of some PFAS exposure may lead to a variety of adverse health outcomes. These health effects include carcinogenicity (Jalilian et al., 2019), hormonal disruption (Chain et al., 2018), immunotoxicity (Chain et al., 2018), liver function alterations (Gleason et al., 2015), low fetal weight (Wikström et al., 2020), increased lipid level (Steenland et al., 2009), tumor induction (Chain et al., 2018), and obesity (Graber et al., 2021). Exposure level of PFAS can be different depending on where people live or what occupations they are involved in. Also, low levels of exposure over long periods of time may pose different types of health risks. Research on long-term effects of low-level exposure to certain PFAS is still in progress.

Adverse health effects reported in firefighters are like those of other occupational groups and the general population exposed to PFAS, including risks for certain cancers (Goodrich et al., 2021). Given the higher rates of certain types of cancer and cancer-related deaths among firefighters, several studies have examined the associations between firefighters’ occupational exposures and cancer incidence (Jalilian et al., 2019; Soteriades et al., 2019). Results have been inconsistent but generally suggest an increased risk of some cancer types such as colon, prostate, and testicular cancers (Soteriades et al., 2019). Besides PFAS, firefighters are exposed to a number of chemical agents, some of which are known carcinogens such as benzene and benzo [a]pyrene (Guerreiro et al., 2016). Little is known about the potential adverse effects of chronic exposure to such complex mixtures. Most of the existing studies so far have focused on health outcomes of individual perfluorinated compounds, with a few exploring their combined effects (Ojo et al., 2020; Ojo et al., 2021). To mimic real-life exposure and for accurate risk assessment, future research focus needs to move to the investigation of such complex mixtures of chemicals instead of single chemicals. However, one of the challenges of mixture risk assessment is the possible interaction between chemicals (i.e., synergistic or antagonistic effects) that may influence the combined activity.

Although a growing body of literature suggests a link between increased serum PFAS levels and cancer incidences, the carcinogenic mechanisms of PFAS are yet to be fully understood. A possible epigenetic mechanism is that occupational exposures in firefighters changes DNA methylation, a process that plays an important role in the healthy regulation of gene expression (Zhou et al., 2019; Goodrich et al., 2021). Changes in DNA methylation pattern can cause inactivation of certain tumor-suppressor genes and thus increase cancer risk (Zhou et al., 2019). In recent years, an increasing number of studies have examined epigenetic changes associated with PFAS exposure. Epigenetics studies focus on alterations in gene expression with no changes in DNA sequence resulting from environmental factors such as chemical exposure (Kim et al., 2021). DNA methylation, histone modification, and microRNA (miRNA) expression are three categories of epigenetic mechanism (Kim et al., 2021). PFAS-induced metabolic alteration is another proposed mechanism for the pro-carcinogenic actions of PFAS. Metabolic reprogramming is an important cancer hallmark (Phan et al., 2014). PFAS can interfere with the body’s metabolic processes and induce biochemical and physiological changes (Jiang et al., 2015; Imir et al., 2021). Due to having structural similarity with fatty acids, PFAS can alter systemic metabolisms by binding to fatty acid transporters and metabolic enzymes (Jiang et al., 2015; Roth et al., 2020; Imir et al., 2021). Both animal and human studies have found evidence of PFAS-induced adverse metabolic effects (Knox et al., 2011; Geiger et al., 2014; Yu et al., 2016; Alderete et al., 2019).

Considering the liver is a primary target organ for long-chain PFAS storage, some researchers have studied the influence of PFAS in human liver cells (Ojo et al., 2020; Ojo et al., 2021). *In vitro* study results have shown dose-dependent association between PFOA exposure and altered DNA methylation (Tian et al., 2012). Other studies highlighted oxidative stress as the possible cause for epigenetic modification. PFOS exposure to hepatic (liver) cells reduced cellular activity and increased reactive oxygen species (ROS) levels in a concentration-dependent manner (Ojo et al., 2020). However, tested doses of PFAS were higher than levels found in the environment indicating these studies may be poor predictors of human reactions to PFAS exposure. Different animal models have also studied the carcinogenic activity of some PFAS. Exposure to PFOA in rodent models was found to be associated with the development of tumor cells in liver, pancreas, and testicles (Steenland and Winquist, 2021). Likewise, studies in rainbow trout observed PFOA exposure promoted the development of liver tumors (Steenland and Winquist, 2021). A recent study by the National Toxicology Program (NTP) found evidence of malignant liver tumor formation in male rats (Sprague-Dawley) induced by PFOA exposure (Program, 2020). The observed association between plasma concentrations of PFOA and tumor incidence suggested the potential link between high PFAS level in blood serum and increased cancer risks (Program, 2020). Similarly, exposure to PFOS in Albino Wistar rat liver showed PFOS-induced changes in miRNA expression and association with liver carcinogenesis (Wang et al., 2012). Although these animal studies provide support for the potential cancer development process, these mechanisms may not appear as relevant in humans.

In addition, PFOA and PFOS-focused work may not give a comprehensive understanding of the relationships between PFAS exposure and firefighter cancers. In this regard, human epidemiological studies can avoid such uncertainties associated with interspecies extrapolation. Several epidemiological studies suggested an association between high-level PFAS in blood serum and increased risk of cancers (Steenland et al., 2020; Bartell and Vieira, 2021). A recent meta-analysis reported that per 10 ng/mL increase in serum PFOA increases the average risk for kidney and testicular cancers, 16% and 3%, respectively (Bartell and Vieira, 2021). Another study concluded that the epidemiologic evidence remains supportive but not definitive for PFOA exposure and kidney and testicular cancer incidences (Steenland et al., 2020). Other epidemiologic studies have shown evidence of PFAS-induced epigenetic changes in both adult populations and birth cohorts (Kim et al., 2021). However, the number of studies is limited when it comes to the context of firefighters’ exposure. One epigenetic study reported an association between years of firefighting and altered DNA methylation (Zhou et al., 2019). The study also observed DNA methylation varied among non-smoker male incumbent firefighters and new recruits (Zhou et al., 2019). Altered miRNA expression has also been linked to PFAS exposure associated with years of firefighting (Jeong et al., 2018). Epigenetic changes are part of the process that leads to cancer (Lu et al., 2020). PFAS-induced epigenetic changes could thus serve as a biomarker to predict the potential health effects in the exposed firefighter community. Given the unpredictability and challenges of fire scenes, monitoring firefighters’ exposure is very complicated. Recent focus has shifted to biomonitoring which may overcome some of the challenges and could serve as a valuable tool for health effects assessment. In this case, the level of biomarkers may vary depending on factors such as pre-existing health conditions (Jabeen et al., 2020), smoking habits (Soteriades et al., 2019), and second job exposure (Soteriades et al., 2019). Future studies should consider these factors while examining the association between firefighters’ PFAS exposure and cancer incidence.

## 8 Challenges of PFAS in hazard assessment

Determining the health risk assessment of PFAS and their precursors is difficult. This is because 1) PFAS are a large, diverse group of substances which inhibits the easy distinction for assessment and management. This makes understanding which PFAS may be relevant for potential human health risk assessment difficult. 2) Very limited information is available on PFAS toxicity and its effects on public health, which makes the chemical-specific evaluation of the diverse PFAS nearly impossible. 3) Humans are frequently exposed to unknown mixtures of PFAS which may cause synergistic effects. 4) Toxicity studies often lack similarities between assays in animals and observation in humans, which makes the relevance of these studies on toxicity uncertain (Anderson et al., 2022).

Grouping of PFAS for mixture assessment is a challenge due to the complexities in the database and differences in regulatory guidance between countries. Hazard assessments for PFAS are usually based on research studies that include representative lead compounds, such as PFOA, PFOS, PFHxS, for which chemical, toxicity, and occurrence information is available (Colnot and Dekant, 2021). There are critical gaps in the understanding of PFAS chemistries and toxicities that inhibit the approach of standard mixtures risk assessment. There is a substantial variation within a PFAS class in their physico-chemical properties. The diversity in their chemical structures, applications, and subsequently their release in the environment and exposure together make the exposure-hazard assessment model very complex. Kwiatkowski et al. (2020) suggested that PFAS should be considered as a single class, and the risk assessment should be performed accordingly (Kwiatkowski et al., 2020).

## 9 Conclusion

PFAS are extensively used in firefighters’ turnout gear, AFFF, and are also present in air and dust of the fire scene and fire station. Therefore, the risk of PFAS exposure is higher for firefighters compared to general population due to the occupational activities during firefighting. Increased cancer rate among the firefighters compared to the general population, and links between PFAS and cancer has raised the concern of PFAS exposure in the fire service. Turnout gear could be a potential source of PFAS exposure as PFCAs and FTOHs may be released from the turnout gear via degradation of water and oil repellent finishes or from release of PFAS contamination for the fire scene. These non-polymeric PFAS could then be potentially absorbed through inhalation, ingestion, and dermal absorption into the firefighter’s body. The ePTFE used in the moisture barrier of the turnout gear is usually considered as a low concern polymer. However, the manufacturing process of ePTFE uses low molecular weight PFAS as polymer processing aids. Therefore, depending on the adulteration, PTFE polymer still could pose adverse health effects by releasing non-polymeric PFAS. Although turnout gear has been identified as a source of PFAS, more research is required to evaluate the PFAS exposure level from turnout gear to firefighters. Besides that, turnout gear may get contaminated by PFAS from the smoke of a structural fire or PFAS-containing AFFF which subsequently act as a source of exposure. Using AFFF during fire extinguishment has been a major source of PFAS exposure for firefighters and the public due to groundwater contamination. The adverse effects of using PFOA and PFOS in AFFF has already forced manufacturers to move to shorter-chain alternative PFAS and more recently fluorine-free alternatives. However, short-chain alternatives may still have adverse health effects and ecotoxicity that cannot be overlooked. Unknown fluorinated components of AFFF and their degradation products still need to be identified. Dust inside fire stations may act as a chemical reservoir of PFAS once compounds leach out from contaminated products. Therefore, indoor air and dust inside fire stations contain high concentrations of PFAS originating from contaminated gear, AFFF usage, or other PFAS-containing products that are present inside the fire station. Other sources of PFAS inside fire stations need to be further explored to achieve a PFAS-free environment in fire stations. Ingestion and inhalation of indoor dust and air are common PFAS exposure pathways. Dermal absorption may also be a dominant exposure pathway for firefighters which takes place due to skin exposure to PFAS contaminated sources. To date, only a few targeted PFAS have been analyzed to determine the risk of dermal absorption. Extensive research is required to better understand dermal absorption of other PFAS compounds. The high level of PFAS exposure at the workplace over a long period of time may increase the risk of firefighters developing health-related issues, including cancer. However, the complex exposure patterns of PFAS coming from multiple sources make it challenging to predict associated risks. Future studies need to address the interactions of PFAS mixtures while evaluating their potential toxicity and health outcomes. Additionally, researchers could leverage epigenetic studies to characterize firefighters’ occupational exposure and their association with the development of work-related diseases. Firefighters are suggested to limit the exposures to PFAS as much as possible. The firefighting community has already started using PFAS-free outer shell materials for their turnout gear and have started transitioning to fluorine-free foams. However, intentionally added PFAS in turnout gear and foams may not be the only sources of occupational PFAS exposure that firefighters experience. Given the volume of PFAS used in consumer products, electronics, building materials, structures, and vehicles it is feasible that these chemicals can be released during combustion and lead to additional exposures to firefighters responding to the incident. These exposures may include respiratory hazards, direct dermal contact, or though settling on and contaminating the turnout gear, as has been shown with multiple other fireground contaminants and carcinogens such as polycyclic aromatic hydrocarbons. Therefore, a general recommendation is not to wear turnout gear where it is not needed (i.e., medical call, personal use, certain types of training, etc.). Also, decontamination should be the done carefully after every fire call to ensure fire stations are not contaminated by the fire scene carcinogens.

## Figures and Tables

**TABLE 1:** List of the PFAS according to their categories and subgroups [adapted from (Panieri et al., 2022)].

	Perfluorinated		Polyfluorinated	
	Subgroup	Example	Subgroup	Example
Non-polymeric PFAS	Perfluoroalkyl acids (PFAAs) Perfluoroalkane sulfonic acids & sulfonates (PFSAs) Perfluoroalkane sulfinic acids (PFSIAs) Perfluorocarboxylic acids & carboxylates (PFCAs) Perfluoroalkyl phosphonic acids (PFPAs) Perfluoroalkyl phosphinic acids (PFPIAs)	PFBS, PFHxS, PFOS PFOSI PFBA, PFHXa, PFOA C8-PFPA C8/C8- PFPiA	Fluorotelomer compounds (FT)	6:2 FTO, 8:2 FTI
			Perfluoroalkane sulfonamido compounds (Me/Et/Bu-FASAs Miscellaneous)	MeFOSA, FOSE 4,8-Dioxa-3H-perfluorononanoate
	Perfluoroalkyl ether acids (PFEAs)	GenX, Adona, F-53B		
	Perfluoroalkane sulfonamides (FASA)	FOSA		
	Perfluoroalkane sulfonyl fluorides (PASFs)	PBSF, POSF		
	Perfluoroalkyl iodides (PFAIs)	PFHxI		
	Perfluoroalkanonyl fluorides (PAFs)	POF		
	Perfluoroalkyl aldehydes (PFALs)	PENAL		
Polymeric PFAS	**Subgroup**		**Example**	
	Fluoropolymers		PVDF, FEP, PFA, ETFE, PTFE	
	Side-chain Fluorinated Polymers		Fluorinated urethane/acrylate/methacrylate/oxetane polymers	
	Perfluoropolyethers (PFPEs)		PEPE-BP, Fluorolink-PFPE	

**TABLE 2:** Estimated elimination Half-lives.

Perfluoroalkyls	Estimated elimination half-lives
PFOA	2.-10.1 years
PFOS	3.3–27 years
PFHxS	4.7–35 years
PFNA	2.5–4.3 years
PFBS	665 h
PFBA	72–81 h

**TABLE 3:** Ionization states of perfluorooctanoic acid.

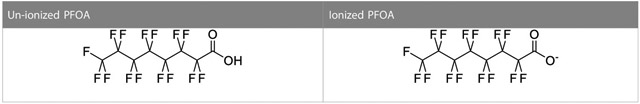

**TABLE 4:** Study PFAS Concentration in Firefighter Blood Serum (geometric mean ng/mL ±95% CI).

	Rotander et al. (2015)	Dobraca et al. (2015)	Trowbridge et al. (2020)	Leary et al. (2020)	Graber et al. (2021)
Sample Year	2013	2010–2011	2014–2015	2018–2019	2019
Cohort Size	149	101	86	36	135
Region	Queensland, Australia	Southern California	San Francisco	Southwest Ohio	New Jersey
Compound					
Perfluoroheptanoic acid (PFHpA)	0.1	0.13 (±0.02)			
Perfluorooctanoic acid (PFOA)	4.6	3.75 (±0.38)	1.15 (±0.10)	2.15	2.07 (±0.18)
Perfluorononanoic acid (PFNA)	0.76	1.15 (±0.10)	0.67 (±0.06)	0.46	0.97 (±0.08)
Perfluorodecanoic acid (PFDA)	0.29	0.90 (±0.12)	0.25 (±0.02)		0.31 (±0.02)
Perfluoroundecanoic acid (PFUnDA)	0.16	0.24 (±0.03)	0.18 (±0.04)		0.11 (±0.01)
Perfluorododecanoic acid (PFDoA)	NC		NC		0.14 (±0.01)
Perfluorobutane sulfonic acid (PFBS)	NC	NC	0.13 (±0.03)		
Perfluorohexane sulfonic acid (PFHxS)	33	2.26 (±0.26)	3.79 (±0.55)	6.15	1.83 (±0.22)
Perfluorooctane sulfonic (PFOS)	74	12.5 (±1.16)	4.11 (±0.43)	8.63	4.25 (±0.55)
Perfluoroactane sulfonamide (PFOSA)		0.032 (±0.005)			
2-(*N*-methyl-PFOSA) acetic acid (MeFOSAA)		0.16 (±0.03)			0.08 (±0.01)
2-(*N*-ethyl-PFOSA) acetic acid (EtFOSAA)		0.016 (±0.002)			

NC—Not calculated due to low detections.

## References

[R1] Abraham K, and Monien BH (2022). Transdermal absorption of ^13^C_4_-perfluorooctanoic acid (^13^C_4_-PFOA) from a sunscreen in a male volunteer–What could be the contribution of cosmetics to the internal exposure of perfluoroalkyl substances (PFAS). Environ. Int 169, 107549. doi:10.1016/j.envint.2022.107549 36191486

[R2] Ahn YS, Jeong KS, and Kim KS (2012). Cancer morbidity of professional emergency responders in Korea. Am. J. Industrial Med. 55 (9), 768–778. doi:10.1002/ajim.22068 22628010

[R3] Alderete TL, Jin R, Walker DI, Valvi D, Chen Z, Jones DP, (2019). Perfluoroalkyl substances, metabolomic profiling, and alterations in glucose homeostasis among overweight and obese hispanic children: A proof-of-concept analysis. Environ. Int 126, 445–453. doi:10.1016/j.envint.2019.02.047 30844580 PMC6555482

[R4] Alexander BM, and Baxter CS (2016). Flame-retardant contamination of firefighter personal protective clothing–a potential health risk for firefighters. J. Occup. Environ. Hyg 13 (9), D148–D155. doi:10.1080/15459624.2016.1183016 27171467

[R5] Anderson J, Brecher R, Cousins IT, DeWitt J, Fiedler H, Kannan K, (2022). Grouping of PFAS for human health risk assessment: Findings from an independent panel of experts. Regul. Toxicol. Pharmacol 134, 105226. doi:10.1016/j.yrtph.2022.105226 35817206

[R6] Appel KE, Gundert-Remy U, Fischer H, Faulde M, Mross KG, Letzel S, (2008). Risk assessment of Bundeswehr (German Federal Armed Forces) permethrin-impregnated battle dress uniforms (BDU). Int. J. Hyg. Environ. health 211 (1-2), 88–104. doi:10.1016/j.ijheh.2007.10.005 18222725

[R7] Audenaert F, Lens H, Rolly D, and Vander Elst P (1999). Fluorochemical textile repellents—synthesis and applications: A 3M perspective. J. Text. Inst 90 (3), 76–94. doi:10.1080/00405009908659480

[R8] Backe WJ, Day TC, and Field JA (2013). Zwitterionic, cationic, and anionic fluorinated chemicals in aqueous film forming foam formulations and groundwater from US military bases by nonaqueous large-volume injection HPLC-MS/MS. Environ. Sci. Technol 47 (10), 5226–5234. doi:10.1021/es3034999 23590254

[R9] Baduel C, Mueller JF, Rotander A, Corfield J, and Gomez-Ramos M-J (2017). Discovery of novel per-and polyfluoroalkyl substances (PFASs) at a fire fighting training ground and preliminary investigation of their fate and mobility. Chemosphere 185, 1030–1038. doi:10.1016/j.chemosphere.2017.06.096 28763938

[R10] Bank R, Smart B, and Tatlow J (1994). Organofluorine chemistry: Principles and commercial application. New York: Plenum and Elsevier.

[R11] Banks APW, Engelsman M, He C, Wang X, and Mueller JF (2020). The occurrence of PAHs and flame-retardants in air and dust from Australian fire stations. J. Occup. Environ. Hyg 17 (2-3), 73–84. doi:10.1080/15459624.2019.1699246 31910147

[R12] Bartell SM, and Vieira VM (2021). Critical review on PFOA, kidney cancer, and testicular cancer. J. Air & Waste Manag. Assoc 71 (6), 663–679. doi:10.1080/10962247.2021.1909668 33780327

[R13] Barzen-Hanson KA, Roberts SC, Choyke S, Oetjen K, McAlees A, Riddell N, (2017). Discovery of 40 classes of per-and polyfluoroalkyl substances in historical aqueous film-forming foams (AFFFs) and AFFF-impacted groundwater. Environ. Sci. Technol 51 (4), 2047–2057. doi:10.1021/acs.est.6b05843 28098989

[R14] Bates MN (2007). Registry-based case–control study of cancer in California firefighters. Am. J. Industrial Med 50 (5), 339–344. doi:10.1002/ajim.20446 17427202

[R15] Beecher N, and Brown S (2018). PFAS and organic residuals management. BioCycle 59, 20.

[R16] Benskin JP, Ikonomou MG, Gobas FA, Begley TH, Woudneh MB, and Cosgrove JR (2013). Biodegradation of N-ethyl perfluorooctane sulfonamido ethanol (EtFOSE) and EtFOSE-based phosphate diester (SAmPAP diester) in marine sediments. Environ. Sci. Technol 47 (3), 1381–1389. doi:10.1021/es304336r 23305554

[R17] Blum A, Balan SA, Scheringer M, Trier X, Goldenman G, Cousins IT, (2015). The Madrid statement on poly-and perfluoroalkyl substances (PFASs). Environ. health Perspect 123 (5), A107–A111. doi:10.1289/ehp.1509934 25932614 PMC4421777

[R18] Blum A, Gold MD, Ames BN, Kenyon C, Jones FR, Hett EA, (1978). Children absorb tris-BP flame retardant from sleepwear: Urine contains the mutagenic metabolite, 2, 3-dibromopropanol. Science 201 (4360), 1020–1023. doi:10.1126/science.684422 684422

[R19] Bonefeld-Jorgensen EC, Long M, Bossi R, Ayotte P, Asmund G, Krüger T, (2011). Perfluorinated compounds are related to breast cancer risk in Greenlandic inuit: A case control study. Environ. Health 10 (1), 88–16. doi:10.1186/1476-069x-10-88 21978366 PMC3203030

[R20] Boyd RI, Ahmad S, Singh R, Fazal Z, Prins GS, Madak Erdogan Z, (2022). Toward a mechanistic understanding of poly-and perfluoroalkylated substances and cancer. Cancers 14 (12), 2919. doi:10.3390/cancers14122919 35740585 PMC9220899

[R21] Brandsma S, Koekkoek J, Van Velzen M, and de Boer J (2019). The PFOA substitute GenX detected in the environment near a fluoropolymer manufacturing plant in The Netherlands. Chemosphere 220, 493–500. doi:10.1016/j.chemosphere.2018.12.135 30594801

[R22] Brown FR, Whitehead TP, Park J-S, Metayer C, and Petreas MX (2014). Levels of non-polybrominated diphenyl ether brominated flame retardants in residential house dust samples and fire station dust samples in California. Environ. Res 135, 9–14. doi:10.1016/j.envres.2014.08.022 25261858 PMC4262617

[R23] Buck RC, Franklin J, Berger U, Conder JM, Cousins IT, De Voogt P, (2011). Perfluoroalkyl and polyfluoroalkyl substances in the environment: Terminology, classification, and origins. Integr. Environ. Assess. Manag 7 (4), 513–541. doi:10.1002/ieam.258 21793199 PMC3214619

[R24] Buck RC, Murphy PM, and Pabon M (2012). “Chemistry, properties, and uses of commercial fluorinated surfactants,” in Polyfluorinated chemicals and transformation products (Berlin, Germany: Springer), 1–24.

[R25] Buck RC (2015). “Toxicology data for alternative “short-chain” fluorinated substances,” in Toxicological effects of perfluoroalkyl and polyfluoroalkyl substances (Berlin, Germany: Springer), 451–477.

[R26] Bursian S, Link J, McCarty M, and Simcik M (2020). The subacute toxicity of PFOS and/or PFOA and legacy aqueous film forming foams to Japanese Quail (Coturnix japonica) Chicks. Environ. Toxicol. Chem 40 (3), 695–710. doi:10.1002/etc.4684 32060944

[R27] Calafat Antonia M, Wong L-Y, Zsuzsanna K, Reidy John A, and Needham Larry L (2007). Polyfluoroalkyl chemicals in the U.S. Population: Data from the national health and nutrition examination survey (NHANES) 2003–2004 and comparisons with NHANES 1999–2000. Environ. health Perspect 115 (11), 1596–1602. doi:10.1289/ehp.10598 18007991 PMC2072821

[R28] Cariou R, Veyrand B, Yamada A, Berrebi A, Zalko D, Durand S, (2015). Perfluoroalkyl acid (PFAA) levels and profiles in breast milk, maternal and cord serum of French women and their newborns. Environ. Int 84, 71–81. doi:10.1016/j.envint.2015.07.014 26232143

[R29] Casjens S, Brüning T, and Taeger D (2020). Cancer risks of firefighters: A systematic review and meta-analysis of secular trends and region-specific differences. Int. archives Occup. Environ. health 93 (7), 839–852. doi:10.1007/s00420-020-01539-0 PMC7452930 32306177

[R30] Chain, E. P. o. C. i. t. F., Knutsen HK, Alexander J, Barregård L, Bignami M, Brüschweiler B, (2018). Risk to human health related to the presence of perfluorooctane sulfonic acid and perfluorooctanoic acid in food. EFSA J. 16 (12), e05194. 32625773 10.2903/j.efsa.2018.5194 PMC7009575

[R31] Chain, E. P. o. C. i. t. F., Schrenk D, Bignami M, Bodin L, Chipman JK, del Mazo J, (2020). Risk to human health related to the presence of perfluoroalkyl substances in food. EFSA J. 18 (9), e06223. 32994824 10.2903/j.efsa.2020.6223 PMC7507523

[R32] Chang ET, Adami H-O, Boffetta P, Cole P, Starr TB, and Mandel JS (2014). A critical review of perfluorooctanoate and perfluorooctanesulfonate exposure and cancer risk in humans. Critical Reviews in Toxicology 44, 1–81. doi:10.3109/10408444.2014.905767 24793953

[R33] Chen F, Yin S, Kelly BC, and Liu W (2017). Isomer-specific transplacental transfer of perfluoroalkyl acids: Results from a survey of paired maternal, cord sera, and placentas. Environ. Sci. Technol 51 (10), 5756–5763. doi:10.1021/acs.est.7b00268 28434222

[R34] Colnot T, and Dekant W (2021). Issues in the hazard and risk assessment of perfluoroalkyl substance mixtures. Toxicol. Lett 353, 79–82. doi:10.1016/j.toxlet.2021.10.005 34666112

[R35] Cousins IT, Vestergren R, Wang Z, Scheringer M, and McLachlan MS (2016). The precautionary principle and chemicals management: The example of perfluoroalkyl acids in groundwater. Environ. Int 94, 331–340. doi:10.1016/j.envint.2016.04.044 27337597

[R36] Croce L, Coperchini F, Tonacchera M, Imbriani M, Rotondi M, and Chiovato L (2019). Effect of long-and short-chain perfluorinated compounds on cultured thyroid cells viability and response to TSH. J. Endocrinol. Investigation 42 (11), 1329–1335. doi:10.1007/s40618-019-01062-1 31102255

[R37] Dai Z, Xia X, Guo J, and Jiang X (2013). Bioaccumulation and uptake routes of perfluoroalkyl acids in Daphnia magna. Chemosphere 90 (5), 1589–1596. doi:10.1016/j.chemosphere.2012.08.026 22967930

[R38] Daniels RD, Bertke S, Dahm MM, Yiin JH, Kubale TL, Hales TR, (2015). Exposure–response relationships for select cancer and non-cancer health outcomes in a cohort of US firefighters from San Francisco, Chicago and Philadelphia (1950–2009). Occup. Environ. Med 72 (10), 699–706. doi:10.1136/oemed-2014-102671 25673342 PMC4558385

[R39] Daniels RD, Kubale TL, Yiin JH, Dahm MM, Hales TR, Baris D, (2014). Mortality and cancer incidence in a pooled cohort of US firefighters from San Francisco, Chicago and Philadelphia (1950–2009). Occup. Environ. Med 71 (6), 388–397. doi:10.1136/oemed-2013-101662 24142974 PMC4499779

[R40] Darwin RL, Ottman RE, Norman EL, Gott JE, and Hanauska CP (1995). Foam and the environment: A delicate balance. NFPA J. 89 (3), 67–73.

[R41] Dauchy X, Boiteux V, Bach C, Rosin C, and Munoz J-F (2017). Per- and polyfluoroalkyl substances in firefighting foam concentrates and water samples collected near sites impacted by the use of these foams. Chemosphere 183, 53–61. doi:10.1016/j.chemosphere.2017.05.056 28531559

[R42] Dauchy X, Boiteux V, Colin A, Hémard J, Bach C, Rosin C, (2019). Deep seepage of per-and polyfluoroalkyl substances through the soil of a firefighter training site and subsequent groundwater contamination. Chemosphere 214, 729–737. doi:10.1016/j.chemosphere.2018.10.003 30293026

[R43] De Silva AO, Allard CN, Spencer C, Webster GM, and Shoeib M (2012). Phosphorus-containing fluorinated organics: Polyfluoroalkyl phosphoric acid diesters (diPAPs), perfluorophosphonates (PFPAs), and perfluorophosphinates (PFPIAs) in residential indoor dust. Environ. Sci. Technol 46 (22), 12575–12582. doi:10.1021/es303172p 23102111

[R44] Dechant J (1985). “Handbook of fiber science and technology,” in *Vol.* II. Chemical processing of fibers and fabrics. Functional finishes: Part B. Hg. von Menachem lewin und Stephen B. Sello (New York/Basel: Marcel Dekker).

[R45] Delahunt B, Bethwaite PB, and Nacey JN (1995). Occupational risk lor renal cell carcinoma. A case-control study based on the New Zealand Cancer Registry. Br. J. urology 75 (5), 578–582. doi:10.1111/j.1464-410x.1995.tb07410.x 7613791

[R46] Demers PA, DeMarini DM, Fent KW, Glass DC, Hansen J, Adetona O, (2022). Carcinogenicity of occupational exposure as a firefighter. Lancet Oncol. 23 (8), 985–986. doi:10.1016/s1470-2045(22)00390-4 35780778

[R47] D’Hollander W, Roosens L, Covaci A, Cornelis C, Reynders H, Van Campenhout K, (2010). Brominated flame retardants and perfluorinated compounds in indoor dust from homes and offices in Flanders, Belgium. Chemosphere 81 (4), 478–487. doi:10.1016/j.chemosphere.2010.07.043 20709355

[R48] Dobraca D, Israel L, McNeel S, Voss R, Wang M, Gajek R, (2015). Biomonitoring in California firefighters: Metals and perfluorinated chemicals. J. Occup. Environ. Med 57 (1), 88–97. doi:10.1097/jom.0000000000000307 25563545 PMC4274322

[R49] Domingo JL, and Nadal M (2019). Human exposure to per-and polyfluoroalkyl substances (PFAS) through drinking water: A review of the recent scientific literature. Environ. Res 177, 108648. doi:10.1016/j.envres.2019.108648 31421451

[R50] Driscoll TR, Carey RN, Peters S, Glass DC, Benke G, Reid A, (2016). The Australian work exposures study: Prevalence of occupational exposure to formaldehyde. Ann. Occup. Hyg 60 (1), 132–138. doi:10.1093/annhyg/mev058 26342091

[R51] Egeghy PP, and Lorber M (2011). An assessment of the exposure of Americans to perfluorooctane sulfonate: A comparison of estimated intake with values inferred from NHANES data. J. Expo. Sci. Environ. Epidemiol 21 (2), 150–168. doi:10.1038/jes.2009.73 20145679

[R52] EI Corporation (2014). Assessment of POP criteria for specific short-chain perfluorinated alkyl substances (Prepared for FluoroCouncil, Washington, DC): ENVIRON International Corporation Arlington, VA.

[R53] EPA (2010). Premanufacture notification exemption for polymers; *Amendment of polymer exemption Rule to exclude certain perfluorinated polymers* [Online]. Environmental Protection Agency (EPA). Available: https://www.federalregister.gov/documents/2010/01/27/2010-1477/premanufacture-notification-exemption-for-polymers-amendment-of-polymer-exemption-rule-to-exclude [Accessed 5/25 2022].

[R54] Fasano W, Kennedy G, Szostek B, Farrar D, Ward R, Haroun L, (2005). Penetration of ammonium perfluorooctanoate through rat and human skin *in vitro*. Drug Chem. Toxicol 28 (1), 79–90. doi:10.1081/dct-39707 15720037

[R55] Fent KW, Alexander B, Roberts J, Robertson S, Toennis C, Sammons D, (2017). Contamination of firefighter personal protective equipment and skin and the effectiveness of decontamination procedures. J. Occup. Environ. Hyg 14 (10), 801–814. doi:10.1080/15459624.2017.1334904 28636458

[R56] Fent KW, Eisenberg J, Snawder J, Sammons D, Pleil JD, Stiegel MA, (2014). Systemic exposure to PAHs and benzene in firefighters suppressing controlled structure fires. Ann. Occup. Hyg 58 (7), 830–845. doi:10.1093/annhyg/meu036 24906357 PMC4124999

[R57] Fent KW, and Evans DE (2011). Assessing the risk to firefighters from chemical vapors and gases during vehicle fire suppression. J. Environ. Monit 13 (3), 536–543. doi:10.1039/c0em00591f 21274476

[R58] Fent KW, Evans DE, Booher D, Pleil JD, Stiegel MA, Horn GP, (2015). Volatile organic compounds off-gassing from firefighters’ personal protective equipment ensembles after use. J. Occup. Environ. Hyg 12 (6), 404–414. doi:10.1080/15459624.2015.1025135 25751596

[R59] Fenton SE, Ducatman A, Boobis A, DeWitt JC, Lau C, Ng C, (2021). Per-and polyfluoroalkyl substance toxicity and human health review: Current state of knowledge and strategies for informing future research. Environ. Toxicol. Chem 40 (3), 606–630. doi:10.1002/etc.4890 33017053 PMC7906952

[R60] Fletcher T, Galloway TS, Melzer D, Holcroft P, Cipelli R, Pilling LC, (2013). Associations between PFOA, PFOS and changes in the expression of genes involved in cholesterol metabolism in humans. Environ. Int 57, 2–10. doi:10.1016/j.envint.2013.03.008 23624243

[R61] Fox H, and Zisman W (1950). The spreading of liquids on low energy surfaces. I. polytetrafluoroethylene. J. Colloid Sci 5 (6), 514–531. doi:10.1016/0095-8522(50)90044-4

[R62] Franko J, Meade B, Frasch HF, Barbero AM, and Anderson SE (2012). Dermal penetration potential of perfluorooctanoic acid (PFOA) in human and mouse skin. J. Toxicol. Environ. Health, Part A 75 (1), 50–62. doi:10.1080/15287394.2011.615108 22047163

[R63] Fraser AJ, Webster TF, Watkins DJ, Nelson JW, Stapleton HM, Calafat AM, (2012). Polyfluorinated compounds in serum linked to indoor air in office environments. Environ. Sci. Technol 46 (2), 1209–1215. doi:10.1021/es2038257 22148395 PMC3262923

[R64] Fromme H, Tittlemier SA, Völkel W, Wilhelm M, and Twardella D (2009). Perfluorinated compounds–exposure assessment for the general population in Western countries. Int. J. Hyg. Environ. health 212 (3), 239–270. doi:10.1016/j.ijheh.2008.04.007 18565792

[R65] Gannon SA, Johnson T, Nabb DL, Serex TL, Buck RC, and Loveless SE (2011). Absorption, distribution, metabolism, and excretion of [1-14C]-perfluorohexanoate ([14C]-PFHx) in rats and mice. Toxicology 283 (1), 55–62. doi:10.1016/j.tox.2011.02.004 21349313

[R66] Gao Y, Fu J, Cao H, Wang Y, Zhang A, Liang Y, (2015). Differential accumulation and elimination behavior of perfluoroalkyl acid isomers in occupational workers in a manufactory in China. Environ. Sci. Technol 49 (11), 6953–6962. doi:10.1021/acs.est.5b00778 25927957

[R67] Gargoubi S, Baffoun A, Harzallah OA, Hamdi M, and Boudokhane C (2020). Water repellent treatment for cotton fabrics with long-chain fluoropolymer and its short-chain eco-friendly alternative. J. Text. Inst 111 (6), 835–845. doi:10.1080/00405000.2019.1664796

[R68] Gasiorowski R, Forbes MK, Silver G, Krastev Y, Hamdorf B, Lewis B, (2022). Effect of plasma and blood donations on levels of perfluoroalkyl and polyfluoroalkyl substances in firefighters in Australia: A randomized clinical trial. JAMA Netw. Open 5 (4), e226257. doi:10.1001/jamanetworkopen.2022.6257 35394514 PMC8994130

[R69] Geiger SD, Xiao J, Ducatman A, Frisbee S, Innes K, and Shankar A (2014). The association between PFOA, PFOS and serum lipid levels in adolescents. Chemosphere 98, 78–83. doi:10.1016/j.chemosphere.2013.10.005 24238303

[R70] Gipe R, and Peterson H (1972). “Background: The first Aqueous Film-Forming Foam (AFFF), introduced into the naval fire,” in Report of NRL progress.

[R71] Gleason JA, Post GB, and Fagliano JA (2015). Associations of perfluorinated chemical serum concentrations and biomarkers of liver function and uric acid in the US population (NHANES), 2007–2010. Environ. Res 136, 8–14. doi:10.1016/j.envres.2014.10.004 25460614

[R72] Glüge J, Scheringer M, Cousins IT, DeWitt JC, Goldenman G, Herzke D, (2020). An overview of the uses of per-and polyfluoroalkyl substances (PFAS). Environ. Sci. Process. Impacts 22 (12), 2345–2373. doi:10.1039/d0em00291g 33125022 PMC7784712

[R73] Goldenman G, Fernandes M, Holland M, Tugran T, Nordin A, Schoumacher C, (2019). The cost of inaction: A socioeconomic analysis of environmental and health impacts linked to exposure to PFAS. Copenhagen, Denmark: Nordic Council of Ministers.

[R74] Golka K, and Weistenhöfer W (2008). Fire fighters, combustion products, and urothelial cancer. J. Toxicol. Environ. Health, Part B 11 (1), 32–44. doi:10.1080/10937400701600396 18176886

[R75] Gonzalez D, Thompson K, Quinones O, Dickenson E, and Bott C (2021). Assessment of PFAS fate, transport, and treatment inhibition associated with a simulated AFFF release within a WASTEWATER treatment plant. Chemosphere 262, 127900. doi:10.1016/j.chemosphere.2020.127900 32799152

[R76] Goodrich JM, Calkins MM, Caban-Martinez AJ, Stueckle T, Grant C, Calafat AM, (2021). Per-and polyfluoroalkyl substances, epigenetic age and DNA methylation: A cross-sectional study of firefighters. Epigenomics 13 (20), 1619–1636. doi:10.2217/epi-2021-0225 34670402 PMC8549684

[R77] Graber JM, Alexander C, Laumbach RJ, Black K, Strickland PO, Georgopoulos PG, (2019). Per and polyfluoroalkyl substances (PFAS) blood levels after contamination of a community water supply and comparison with 2013–2014 NHANES. J. Expo. Sci. Environ. Epidemiol 29 (2), 172–182. doi:10.1038/s41370-018-0096-z 30482936 PMC6380951

[R78] Graber JM, Black TM, Shah NN, Caban-Martinez AJ, Lu S. e., Brancard T, (2021). Prevalence and predictors of per-and polyfluoroalkyl substances (PFAS) serum levels among members of a suburban us volunteer fire department. Int. J. Environ. Res. public health 18 (7), 3730. doi:10.3390/ijerph18073730 33918459 PMC8038206

[R79] Grajeck E, and Peterson W (1959). Fluorochemicals: The new idea in textiles. Am. Dyest. Rep 48 (13), 37–39.

[R80] Grandjean P, and Clapp R (2015). Perfluorinated alkyl substances: Emerging insights into health risks. New solutions a J. Environ. Occup. health policy 25 (2), 147–163. doi:10.1177/1048291115590506 PMC6172956 26084549

[R81] Gremmel C, Frömel T, and Knepper TP (2016). Systematic determination of perfluoroalkyl and polyfluoroalkyl substances (PFASs) in outdoor jackets. Chemosphere 160, 173–180. doi:10.1016/j.chemosphere.2016.06.043 27376856

[R82] Gribble MO, Bartell SM, Kannan K, Wu Q, Fair PA, and Kamen DL (2015). Longitudinal measures of perfluoroalkyl substances (PFAS) in serum of gullah african Americans in South Carolina: 2003–2013. Environ. Res 143, 82–88. doi:10.1016/j.envres.2015.03.012 25819541 PMC4583839

[R83] Guerreiro C, Horálek J, de Leeuw F, and Couvidat F (2016). Benzo (a) pyrene in Europe: Ambient air concentrations, population exposure and health effects. Environ. Pollut 214, 657–667. doi:10.1016/j.envpol.2016.04.081 27140679

[R84] Guo J, Resnick P, Efimenko K, Genzer J, and DeSimone JM (2008). Alternative fluoropolymers to avoid the challenges associated with perfluorooctanoic acid. Industrial Eng. Chem. Res 47 (3), 502–508. doi:10.1021/ie0703179

[R85] Gützkow KB, Haug LS, Thomsen C, Sabaredzovic A, Becher G, and Brunborg G (2012). Placental transfer of perfluorinated compounds is selective–a Norwegian Mother and Child sub-cohort study. Int. J. Hyg. Environ. health 215 (2), 216–219. doi:10.1016/j.ijheh.2011.08.011 21937271

[R86] Hall SM, Patton S, Petreas M, Zhang S, Phillips AL, Hoffman K, (2020). Per-and polyfluoroalkyl substances in dust collected from residential homes and fire stations in North America. Environ. Sci. Technol 54 (22), 14558–14567. doi:10.1021/acs.est.0c04869 33143410 PMC7939574

[R87] Han J-S, Jang S, Son H-Y, Kim Y-B, Kim Y, Noh J-H, (2020). Subacute dermal toxicity of perfluoroalkyl carboxylic acids: Comparison with different carbon-chain lengths in human skin equivalents and systemic effects of perfluoroheptanoic acid in sprague dawley rats. Archives Toxicol. 94 (2), 523–539. doi:10.1007/s00204-019-02634-z 31797001

[R88] Harrad S, de Wit CA, Abdallah MA-E, Bergh C, Bjorklund JA, Covaci A, (2010). Indoor contamination with hexabromocyclododecanes, polybrominated diphenyl ethers, and perfluoroalkyl compounds: An important exposure pathway for people? Environ. Sci. Technol 44 (9), 3221–3231. doi:10.1021/es903476t 20387882

[R89] Haug LS, Huber S, Becher G, and Thomsen C (2011a). Characterisation of human exposure pathways to perfluorinated compounds—Comparing exposure estimates with biomarkers of exposure. Environ. Int 37 (4), 687–693. doi:10.1016/j.envint.2011.01.011 21334069

[R90] Haug LS, Huber S, Schlabach M, Becher G, and Thomsen C (2011b). Investigation on per-and polyfluorinated compounds in paired samples of house dust and indoor air from Norwegian homes. Environ. Sci. Technol 45 (19), 7991–7998. doi:10.1021/es103456h 21417377

[R91] Henry BJ, Carlin JP, Hammerschmidt JA, Buck RC, Buxton LW, Fiedler H, (2018). A critical review of the application of polymer of low concern and regulatory criteria to fluoropolymers. Integr. Environ. Assess. Manag 14 (3), 316–334. doi:10.1002/ieam.4035 29424474

[R92] Hill PJ, Taylor M, Goswami P, and Blackburn RS (2017). Substitution of PFAS chemistry in outdoor apparel and the impact on repellency performance. Chemosphere 181, 500–507. doi:10.1016/j.chemosphere.2017.04.122 28460297

[R93] Høisæter Å, Pfaff A, and Breedveld GD (2019). Leaching and transport of PFAS from aqueous film-forming foam (AFFF) in the unsaturated soil at a firefighting training facility under cold climatic conditions. J. Contam. hydrology 222, 112–122. doi:10.1016/j.jconhyd.2019.02.010 30878240

[R94] Holmes DA (2000). “Waterproof breathable fabrics,” in Handbook of technical textiles, 282315. Cambridge, England: Woodhead Publishing Limited.

[R95] Holmquist H, Schellenberger S, van Der Veen I, Peters G, Leonards P, and Cousins IT (2016). Properties, performance and associated hazards of state-of-the-art durable water repellent (DWR) chemistry for textile finishing. Environ. Int 91, 251–264. doi:10.1016/j.envint.2016.02.035 26994426

[R96] Holzapfel W (1966). Uses of fluorinated chemicals. Fette, Seifen, Anstrichm. 68 (10), 837–842.

[R97] Honda K, Morita M, Otsuka H, and Takahara A (2005). Molecular aggregation structure and surface properties of poly (fluoroalkyl acrylate) thin films. Macromolecules 38 (13), 5699–5705. doi:10.1021/ma050394k

[R98] Hopkins ZR, Sun M, DeWitt JC, and Knappe DR (2018). Recently detected drinking water contaminants: GenX and other per-and polyfluoroalkyl ether acids. Journal-American Water Works Assoc. 110 (7), 13–28. doi:10.1002/awwa.1073

[R99] Houtz EF, Higgins CP, Field JA, and Sedlak DL (2013). Persistence of perfluoroalkyl acid precursors in AFFF-impacted groundwater and soil. Environ. Sci. Technol 47 (15), 8187–8195. doi:10.1021/es4018877 23886337

[R100] Huang H, Yu K, Zeng X, Chen Q, Liu Q, Zhao Y, (2020). Association between prenatal exposure to perfluoroalkyl substances and respiratory tract infections in preschool children. Environ. Res 191, 110156. doi:10.1016/j.envres.2020.110156 32871147

[R101] Hulett DM, Bendick M, Sheila Y, and Thomas F (2007). Enhancing women’s inclusion in firefighting. Washington, D.C: Bendick and Egan Economic Consultants, Inc.

[R102] IARC Working Group on the Evaluation of Carcinogenic Risks to Humans (2010). Painting, firefighting, and shiftwork. IARC Monogr. Eval. Carcinog. risks humans 98, 9–764. PMC4781497 21381544

[R103] Imir OB, Kaminsky AZ, Zuo Q-Y, Liu Y-J, Singh R, Spinella MJ, (2021). Per-and polyfluoroalkyl substance exposure combined with high-fat diet supports prostate cancer progression. Nutrients 13 (11), 3902. doi:10.3390/nu13113902 34836157 PMC8623692

[R104] Inoue K, Okada F, Ito R, Kato S, Sasaki S, Nakajima S, (2004). Perfluorooctane sulfonate (PFOS) and related perfluorinated compounds in human maternal and cord blood samples: Assessment of PFOS exposure in a susceptible population during pregnancy. Environ. health Perspect 112 (11), 1204–1207. doi:10.1289/ehp.6864 15289168 PMC1247483

[R105] Islam MT, Rahman MM, and Mazumder NUS (2020). “Polymers for textile production,” in Frontiers of textile materials: Polymers, nanomaterials, enzymes, and advanced modification techniques, 13–59. New York, USA: John Wiley & Sons, Ltd.

[R106] IW Group (2016). “IARC working group international agency for research on cancer volume 117: Pentachlorophenol and some related compounds, lyon; 4–11 october 2016,” in IARC monogr. Eval. Carcinog. Risk chem, hum.

[R107] Jabeen M, Fayyaz M, and Irudayaraj J (2020). Epigenetic modifications, and alterations in cell cycle and apoptosis pathway in A549 lung carcinoma cell line upon exposure to perfluoroalkyl substances. Toxics 8 (4), 112. doi:10.3390/toxics8040112 33238432 PMC7711517

[R108] Jain RB, and Ducatman A (2019). Selective associations of recent low concentrations of perfluoroalkyl substances with liver function biomarkers: NHANES 2011 to 2014 data on US adults aged≥ 20 years. J. Occup. Environ. Med 61 (4), 293–302. doi:10.1097/jom.0000000000001532 30589657

[R109] Jalilian H, Ziaei M, Weiderpass E, Rueegg CS, Khosravi Y, and Kjaerheim K (2019). Cancer incidence and mortality among firefighters. Int. J. cancer 145 (10), 2639–2646. doi:10.1002/ijc.32199 30737784

[R110] Jeong KS, Zhou J, Griffin SC, Jacobs ET, Dearmon-Moore D, Zhai J, (2018). MicroRNA changes in firefighters. J. Occup. Environ. Med 60 (5), 469–474. doi:10.1097/jom.0000000000001307 29465512 PMC5959213

[R111] Jiang Q, Gao H, and Zhang L (2015). “Metabolic effects PFAS,” in Toxicological effects of perfluoroalkyl and polyfluoroalkyl substances, 177–201. Cham, New Jersey, USA: Humana Press.

[R112] Jin C, Sun Y, Islam A, Qian Y, and Ducatman A (2011). Perfluoroalkyl acids including perfluorooctane sulfonate and perfluorohexane sulfonate in firefighters. J. Occup. Environ. Med 53, 324–328. doi:10.1097/jom.0b013e31820d1314 21346631

[R113] Jochyms Q, Mignard E, and Vincent J-M (2015). Fluorosurfactants for applications in catalysis. J. Fluor. Chem 177, 11–18. doi:10.1016/j.jfluchem.2015.01.011

[R114] Johnson MS, Buck RC, Cousins IT, Weis CP, and Fenton SE (2021). Estimating environmental hazard and risks from exposure to per-and polyfluoroalkyl substances (PFASs): Outcome of a SETAC focused topic meeting. New York, United States: Wiley Online Library. 10.1002/etc.4784 PMC8387100 32452041

[R115] Jones L, Burgess JL, Evans H, and Lutz EA (2016). Respiratory protection for firefighters—evaluation of CBRN canisters for use during overhaul II: In mask analyte sampling with integrated dynamic breathing machine. J. Occup. Environ. Hyg 13 (3), 177–184. doi:10.1080/15459624.2015.1091964 26554925

[R116] Jones PD, Hu W, De Coen W, Newsted JL, and Giesy JP (2003). Binding of perfluorinated fatty acids to serum proteins. Environ. Toxicol. Chem. An Int. J 22 (11), 2639–2649. doi:10.1897/02-553 14587903

[R117] Jones-Otazo HA, Clarke JP, Diamond ML, Archbold JA, Ferguson G, Harner T, (2005). Is house dust the missing exposure pathway for PBDEs? An analysis of the urban fate and human exposure to PBDEs. Environ. Sci. Technol 39 (14), 5121–5130. doi:10.1021/es048267b 16082939

[R118] Kang D, Davis LK, Hunt P, and Kriebel D (2008). Cancer incidence among male Massachusetts firefighters, 1987–2003. Am. J. Industrial Med 51 (5), 329–335. doi:10.1002/ajim.20549 18306327

[R119] Keir JLA, Akhtar US, Matschke DMJ, White PA, Kirkham TL, Chan HM, (2020). Polycyclic aromatic hydrocarbon (PAH) and metal contamination of air and surfaces exposed to combustion emissions during emergency fire suppression: Implications for firefighters’ exposures. Sci. total Environ 698, 134211. doi:10.1016/j.scitotenv.2019.134211 31514022

[R120] Kempisty DM, and Racz L (2021). Forever chemicals: Environmental, economic, and social equity concerns with PFAS in the environment. Boca Raton, Florida: CRC Press.

[R121] Kempisty DM, Xing Y, and Racz L (2016). Perfluoroalkyl substances in the environment: Theory, practice, and innovation.

[R122] Kim S, Thapar I, and Brooks BW (2021). Epigenetic changes by per-and polyfluoroalkyl substances (PFAS). Environ. Pollut 279, 116929. doi:10.1016/j.envpol.2021.116929 33751946

[R123] Kissa E (2001). Fluorinated surfactants and repellents. Boca Raton, Florida: CRC Press.

[R124] Kissa E (1994). Fluorinated surfactants, Surfactant science series, 50. New York: Marcel Dekker.

[R125] Knox SS, Jackson T, Javins B, Frisbee SJ, Shankar A, and Ducatman AM (2011). Implications of early menopause in women exposed to perfluorocarbons. J. Clin. Endocrinol. Metabolism 96 (6), 1747–1753. doi:10.1210/jc.2010-2401 PMC3206400 21411548

[R126] Korzeniowski SH, Buck RC, Kempisty DM, and Pabon M (2018). “Fluorosurfactants in firefighting foams: Past and present,” in Perfluoroalkyl substances in the environment (Boca Raton, Florida: CRC Press), 3–34.

[R127] Kotlarz N, McCord J, Collier D, Lea CS, Strynar M, Lindstrom AB, (2020). Measurement of novel, drinking water-associated PFAS in blood from adults and children in Wilmington, North Carolina. Environ. health Perspect 128 (7), 077005. doi:10.1289/ehp6837 32697103 PMC7375159

[R128] Kwiatkowski CF, Andrews DQ, Birnbaum LS, Bruton TA, DeWitt JC, Knappe DR, (2020). Scientific basis for managing PFAS as a chemical class. Environ. Sci. Technol. Lett 7 (8), 532–543. doi:10.1021/acs.estlett.0c00255 34307722 PMC8297807

[R129] Laitinen JA, Koponen J, Koikkalainen J, and Kiviranta H (2014). Firefighters’ exposure to perfluoroalkyl acids and 2-butoxyethanol present in firefighting foams. Toxicol. Lett 231 (2), 227–232. doi:10.1016/j.toxlet.2014.09.007 25447453

[R130] Laitinen J, Mäkelä M, Mikkola J, and Huttu I (2012). Firefighters’ multiple exposure assessments in practice. Toxicol. Lett 213 (1), 129–133. doi:10.1016/j.toxlet.2012.06.005 22710199

[R131] Laroche E, and L’Espérance S (2021). Cancer incidence and mortality among firefighters: An overview of epidemiologic systematic reviews. Int. J. Environ. Res. Public Health 18 (5), 2519. doi:10.3390/ijerph18052519 33802629 PMC7967542

[R132] Lau C, Anitole K, Hodes C, Lai D, Pfahles-Hutchens A, and Seed J (2007). Perfluoroalkyl acids: A review of monitoring and toxicological findings. Toxicol. Sci 99 (2), 366–394. doi:10.1093/toxsci/kfm128 17519394

[R133] Leary DB, Takazawa M, Kannan K, and Khalil N (2020). Perfluoroalkyl substances and metabolic syndrome in firefighters: A pilot study. J. Occup. Environ. Med 62 (1), 52–57. doi:10.1097/jom.0000000000001756 31658221

[R134] Lee DJ, Koru-Sengul T, Hernandez MN, Caban-Martinez AJ, McClure LA, Mackinnon JA, (2020). Cancer risk among career male and female Florida firefighters: Evidence from the Florida Firefighter Cancer Registry (1981-2014). Am. J. Industrial Med 63 (4), 285–299. doi:10.1002/ajim.23086 31930542

[R135] LeMasters GK, Genaidy AM, Succop P, Deddens J, Sobeih T, Barriera-Viruet H, (2006). Cancer risk among firefighters: A review and meta-analysis of 32 studies. J. Occup. Environ. Med 48, 1189–1202. doi:10.1097/01.jom.0000246229.68697.90 17099456

[R136] Li Y, Fletcher T, Mucs D, Scott K, Lindh CH, Tallving P, (2018). Half-lives of PFOS, PFHxS and PFOA after end of exposure to contaminated drinking water. Occup. Environ. Med 75 (1), 46–51. doi:10.1136/oemed-2017-104651 29133598 PMC5749314

[R137] Li Y, Xu Y, Fletcher T, Scott K, Nielsen C, Pineda D, (2021). Associations between perfluoroalkyl substances and thyroid hormones after high exposure through drinking water. Environ. Res 194, 110647. doi:10.1016/j.envres.2020.110647 33358873

[R138] Liagkouridis I, Awad R, Schellenberger S, Plassmann MM, Cousins IT, and Benskin JP (2021). Combined use of total fluorine and oxidative fingerprinting for quantitative determination of side-chain fluorinated polymers in textiles. Environ. Sci. Technol. Lett 9 (1), 30–36. doi:10.1021/acs.estlett.1c00822

[R139] Lindstrom AB, Strynar MJ, and Libelo EL (2011). Polyfluorinated compounds: Past, present, and future. Environ. Sci. Technol 45 (19), 7954–7961. doi:10.1021/es2011622 21866930

[R140] Liu C, Gin KY, Chang VW, Goh BP, and Reinhard M (2011). Novel perspectives on the bioaccumulation of PFCs–the concentration dependency. Environ. Sci. Technol 45 (22), 9758–9764. doi:10.1021/es202078n 21988464

[R141] Liu J, and Avendaño SM (2013). Microbial degradation of polyfluoroalkyl chemicals in the environment: A review. Environ. Int 61, 98–114. doi:10.1016/j.envint.2013.08.022 24126208

[R142] Lohmann R, Cousins IT, DeWitt JC, Gluge J, Goldenman G, Herzke D, (2020). Are fluoropolymers really of low concern for human and environmental health and separate from other PFAS? Environ. Sci. Technol 54 (20), 12820–12828. doi:10.1021/acs.est.0c03244 33043667 PMC7700770

[R143] Lu Y, Chan Y-T, Tan H-Y, Li S, Wang N, and Feng Y (2020). Epigenetic regulation in human cancer: The potential role of epi-drug in cancer therapy. Mol. cancer 19 (1), 79–16. doi:10.1186/s12943-020-01197-3 32340605 PMC7184703

[R144] Lu Y, Meng L, Ma D, Cao H, Liang Y, Liu H, (2021). The occurrence of PFAS in human placenta and their binding abilities to human serum albumin and organic anion transporter 4. Environ. Pollut 273, 116460. doi:10.1016/j.envpol.2021.116460 33485002

[R145] Mahltig B (2015). “13 - hydrophobic and oleophobic finishes for textiles,” in Functional finishes for textiles. Editor Paul R (Sawston, United Kingdom: Woodhead Publishing), 387–428.

[R146] Mandal S, Mazumder N-U-S, Agnew RJ, Song G, and Li R (2021). Characterization and modeling of thermal protective and thermo-physiological comfort performance of polymeric textile materials—a review. Materials 14 (9), 2397. doi:10.3390/ma14092397 34062955 PMC8124731

[R147] Marmur A (2006). Soft contact: Measurement and interpretation of contact angles. Soft Matter 2 (1), 12–17. doi:10.1039/b514811c 32646087

[R148] Mastrantonio M, Bai E, Uccelli R, Cordiano V, Screpanti A, and Crosignani P (2018). Drinking water contamination from perfluoroalkyl substances (PFAS): An ecological mortality study in the veneto region, Italy. Eur. J. Public Health 28 (1), 180–185. doi:10.1093/eurpub/ckx066 28541558

[R149] Mayer AC, Fent KW, Bertke S, Horn GP, Smith DL, Kerber S, (2019). Firefighter hood contamination: Efficiency of laundering to remove PAHs and FRs. J. Occup. Environ. Hyg 16 (2), 129–140. doi:10.1080/15459624.2018.1540877 30427284 PMC8647047

[R150] Mazumder N-U-S (2021). Characterizing the tensile strength of outer layer fabrics used in firefighters’ protective clothing under radiant heat exposure. Stillwater, OK, United States: Oklahoma State University.

[R151] Mazumder N-U-S, Mandal S, Agnew RJ, Petrova A, Boorady LM, and Song G (2022). Characterizing the tensile strength of the fabrics used in firefighters’ bunker gear under radiant heat exposure. Polymers 14 (2), 296. doi:10.3390/polym14020296 35054702 PMC8780976

[R152] Mazumder NUS, and Islam MT (2021). “Flame retardant finish for textile fibers,” in Innovative and emerging technologies for textile dyeing and finishing, 373–405. New York, USA: John Wiley & Sons, Ltd.

[R153] McGuire ME, Schaefer C, Richards T, Backe WJ, Field JA, Houtz E, (2014). Evidence of remediation-induced alteration of subsurface poly-and perfluoroalkyl substance distribution at a former firefighter training area. Environ. Sci. Technol 48 (12), 6644–6652. doi:10.1021/es5006187 24866261

[R154] Meegoda JN, Kewalramani JA, Li B, and Marsh RW (2020). A review of the applications, environmental release, and remediation technologies of per-and polyfluoroalkyl substances. Int. J. Environ. Res. public health 17 (21), 8117. doi:10.3390/ijerph17218117 33153160 PMC7663283

[R155] Moody CA, and Field JA (2000). Perfluorinated surfactants and the environmental implications of their use in fire-fighting foams. Environ. Sci. Technol 34 (18), 3864–3870. doi:10.1021/es991359u

[R156] Moya J, Phillips L, Schuda L, Wood P, Diaz A, Lee R, (2011). Exposure factors handbook: 2011 edition. Washington, D.C., United States: US Environmental Protection Agency.

[R157] Muegge CM, Zollinger TW, Song Y, Wessel J, Monahan PO, and Moffatt SM (2018). Excess mortality among Indiana firefighters, 1985-2013. Am. J. Industrial Med 61 (12), 961–967. doi:10.1002/ajim.22918 30421827

[R158] Muensterman DJ, Titaley IA, Peaslee GF, Minc LD, Cahuas L, Rodowa AE, (2021). Disposition of fluorine on new firefighter turnout gear. Washington, DC: Environmental science & technology. 10.1021/acs.est.1c06322 34961317

[R159] Nadal M, and Domingo JL (2014). Indoor dust levels of perfluoroalkyl substances (PFASs) and the role of ingestion as an exposure pathway: A review. Curr. Org. Chem 18 (17), 2200–2208. doi:10.2174/1385272819666140804230713

[R160] Nelson JW, Hatch EE, and Webster TF (2010). Exposure to polyfluoroalkyl chemicals and cholesterol, body weight, and insulin resistance in the general US population. Environ. health Perspect 118 (2), 197–202. doi:10.1289/ehp.0901165 20123614 PMC2831917

[R161] OECD (2009). Data analysis of the identification of correlations between polymer characteristics and potential for health or ecotoxicological concern. Paris: Organisation for Economic Co-Operation and Development.

[R162] OECD (2021). Reconciling terminology of the universe of per- and polyfluoroalkyl substances: Recommendations and practical guidance. [Online]. Paris, France: OECD. Available: https://www.oecd.org/officialdocuments/publicdisplaydocumentpdf/?cote=ENV/CBC/MONO(2021)25&docLanguage=En (Accessed May 23, 2022).

[R163] OECD (2018). Toward a new comprehensive global database of per-and polyfluoroalkyl substances (PFASs): Summary report on updating the OECD 2007 list of per-and polyfluoroalkyl substances (PFASs). Paris: Organisation for Economic Cooperation and Development (OECD).

[R164] Ojo AF, Peng C, and Ng JC (2020). Combined effects and toxicological interactions of perfluoroalkyl and polyfluoroalkyl substances mixtures in human liver cells (HepG2). Environ. Pollut 263, 114182. doi:10.1016/j.envpol.2020.114182 32247900

[R165] Ojo AF, Xia Q, Peng C, and Ng JC (2021). Evaluation of the individual and combined toxicity of perfluoroalkyl substances to human liver cells using biomarkers of oxidative stress. Chemosphere 281, 130808. doi:10.1016/j.chemosphere.2021.130808 34022600

[R166] Oliveira M, Slezakova K, Alves MJ, Fernandes A, Teixeira JP, Delerue-Matos C, (2017). Polycyclic aromatic hydrocarbons at fire stations: Firefighters’ exposure monitoring and biomonitoring, and assessment of the contribution to total internal dose. J. Hazard. Mater 323, 184–194. doi:10.1016/j.jhazmat.2016.03.012 26997333

[R167] Olsen GW, Mair DC, Lange CC, Harrington LM, Church TR, Goldberg CL, (2017). Per-and polyfluoroalkyl substances (PFAS) in American Red Cross adult blood donors, 2000–2015. Environ. Res 157, 87–95. doi:10.1016/j.envres.2017.05.013 28528142

[R168] Pabon M, and Corpart J (2002). Fluorinated surfactants: Synthesis, properties, effluent treatment. J. Fluor. Chem 114 (2), 149–156. doi:10.1016/s0022-1139(02)00038-6

[R169] Panieri E, Baralic K, Djukic-Cosic D, Buha Djordjevic A, and Saso L (2022). PFAS molecules: A major concern for the human health and the environment. Toxics 10 (2), 44. doi:10.3390/toxics10020044 35202231 PMC8878656

[R170] Peaslee GF, Wilkinson JT, McGuinness SR, Tighe M, Caterisano N, Lee S, (2020). Another pathway for firefighter exposure to per-and polyfluoroalkyl substances: Firefighter textiles. Environ. Sci. Technol. Lett 7 (8), 594–599. doi:10.1021/acs.estlett.0c00410

[R171] Peshoria S, Nandini D, Tanwar R, and Narang R (2020). Short-chain and long-chain fluorosurfactants in firefighting foam: A review. Environ. Chem. Lett 18 (4), 1277–1300. doi:10.1007/s10311-020-01015-8

[R172] Phan LM, Yeung S-CJ, and Lee M-H (2014). Cancer metabolic reprogramming: Importance, main features, and potentials for precise targeted anti-cancer therapies. Cancer Biol. Med 11 (1), 1–19. doi:10.7497/j.issn.2095-3941.2014.01.001 24738035 PMC3969803

[R173] Pizzurro DM, Seeley M, Kerper LE, and Beck BD (2019). Interspecies differences in perfluoroalkyl substances (PFAS) toxicokinetics and application to health-based criteria. Regul Toxicol. Pharmacol 106, 239–250. doi:10.1016/j.yrtph.2019.05.008 31078680

[R174] Place BJ, and Field JA (2012). Identification of novel fluorochemicals in aqueous film-forming foams used by the US military. Environ. Sci. Technol 46 (13), 7120–7127. doi:10.1021/es301465n 22681548 PMC3390017

[R175] Plumlee MH, McNeill K, and Reinhard M (2009). Indirect photolysis of perfluorochemicals: Hydroxyl radical-initiated oxidation of N-ethyl perfluorooctane sulfonamido acetate (N-EtFOSAA) and other perfluoroalkanesulfonamides. Environ. Sci. Technol 43 (10), 3662–3668. doi:10.1021/es803411w 19544870

[R176] Poothong S, Papadopoulou E, Padilla-Sánchez JA, Thomsen C, and Haug LS (2020). Multiple pathways of human exposure to poly-and perfluoroalkyl substances (PFASs): From external exposure to human blood. Environ. Int 134, 105244. doi:10.1016/j.envint.2019.105244 31711019

[R177] Porter MR (2013). Handbook of surfactants. Berlin, Germany: Springer.

[R178] Posner S (2012). “Perfluorinated compounds: Occurrence and uses in products,” in Polyfluorinated chemicals and transformation products (Berlin, Germany: Springer), 25–39.

[R179] Post GB, Cohn PD, and Cooper KR (2012). Perfluorooctanoic acid (PFOA), an emerging drinking water contaminant: A critical review of recent literature. Environ. Res 116, 93–117. doi:10.1016/j.envres.2012.03.007 22560884

[R180] Program NT (2020). NTP technical report on the toxicology and carcinogenesis studies of perfluorooctanoic acid (CASRN 335-67-1) administered in feed to Sprague Dawley (Hsd: Sprague Dawley^®^ SD^®^) rats. NC, USA: U.S. Department of Health and Human Services. 10.22427/NTP-TR-598 PMC8039881 33556048

[R181] Ragnarsdóttir O, Abdallah MA-E, and Harrad S (2022). Dermal uptake: An important pathway of human exposure to perfluoroalkyl substances? Environ. Pollut 307, 119478. doi:10.1016/j.envpol.2022.119478 35588958

[R182] Rankin K (2015). Fluorotelomer-based acrylate polymers as an indirect source of perfluoroalkyl carboxylates. Toronto, ON M5S, Canada: University of Toronto.

[R183] Rankin K, Lee H, Tseng PJ, and Mabury SA (2014). Investigating the biodegradability of a fluorotelomer-based acrylate polymer in a soil–plant microcosm by indirect and direct analysis. Environ. Sci. Technol 48 (21), 12783–12790. doi:10.1021/es502986w 25296394

[R184] Rewerts JN, Morré JT, Massey Simonich SL, and Field JA (2018). In-vial extraction large volume gas chromatography mass spectrometry for analysis of volatile PFASs on papers and textiles. Environ. Sci. Technol 52 (18), 10609–10616. doi:10.1021/acs.est.8b04304 30148348

[R185] Rickard BP, Rizvi I, and Fenton SE (2022). Per-and poly-fluoroalkyl substances (PFAS) and female reproductive outcomes: PFAS elimination, endocrine-mediated effects, and disease. Toxicology 465, 153031. doi:10.1016/j.tox.2021.153031 34774661 PMC8743032

[R186] Rizzuto P (2020). Older PFAS That EPA Thought Obsolete Still Used, Agency Told. Available at: https://news.bloomberglaw.com/environment-and-energy/older-pfas-that-epa-thought-obsolete-still-used-agency-told (Accessed December 15, 2022).

[R187] Robel AE, Marshall K, Dickinson M, Lunderberg D, Butt C, Peaslee G, (2017). Closing the mass balance on fluorine on papers and textiles. Environ. Sci. Technol 51 (16), 9022–9032. doi:10.1021/acs.est.7b02080 28712295

[R188] Rosenfeld PE, Spaeth KR, Remy LL, Byers V, Muerth SA, Hallman RC, (2023). Perfluoroalkyl substances exposure in firefighters: Sources and implications. Environ. Res 220, 115164. doi:10.1016/j.envres.2022.115164 36584840

[R189] Rossbach B, Niemietz A, Kegel P, and Letzel S (2014). Uptake and elimination of permethrin related to the use of permethrin treated clothing for forestry workers. Toxicol. Lett 231 (2), 147–153. doi:10.1016/j.toxlet.2014.10.017 25455447

[R190] Rotander A, Toms L-ML, Aylward L, Kay M, and Mueller JF (2015). Elevated levels of PFOS and PFHxS in firefighters exposed to aqueous film forming foam (AFFF). Environ. Int 82, 28–34. doi:10.1016/j.envint.2015.05.005 26001497

[R191] Roth K, Imran Z, Liu W, and Petriello MC (2020). Diet as an exposure source and mediator of per-and polyfluoroalkyl substance (PFAS) toxicity. Front. Toxicol 2, 601149. doi:10.3389/ftox.2020.601149 35296120 PMC8915917

[R192] Russell MH, Berti WR, Szostek B, and Buck RC (2008). Investigation of the biodegradation potential of a fluoroacrylate polymer product in aerobic soils. Environ. Sci. Technol 42 (3), 800–807. doi:10.1021/es0710499 18323105

[R193] Sayed U, and Dabhi P (2014). “Finishing of textiles with fluorocarbons,” in Waterproof and water repellent textiles and clothing (Amsterdam, Netherlands: Elsevier), 139–152.

[R194] Schellenberger S, Gillgard P, Stare A, Hanning A, Levenstam O, Roos S, (2018). Facing the rain after the phase out: Performance evaluation of alternative fluorinated and non-fluorinated durable water repellents for outdoor fabrics. Chemosphere 193, 675–684. doi:10.1016/j.chemosphere.2017.11.027 29172158

[R195] Schellenberger S, Jonsson C, Mellin P, Levenstam OA, Liagkouridis I, Ribbenstedt A, (2019). Release of side-chain fluorinated polymer-containing microplastic fibers from functional textiles during washing and first estimates of perfluoroalkyl acid emissions. Environ. Sci. Technol 53 (24), 14329–14338. doi:10.1021/acs.est.9b04165 31697071

[R196] Schroeder T, Bond D, and Foley J (2021). PFAS soil and groundwater contamination via industrial airborne emission and land deposition in SW Vermont and Eastern New York State, USA. Environ. Sci. Process. Impacts 23 (2), 291–301. doi:10.1039/d0em00427h 33443261

[R197] Schultes L, Peaslee GF, Brockman JD, Majumdar A, McGuinness SR, Wilkinson JT, (2019). Total fluorine measurements in food packaging: How do current methods perform? Environ. Sci. Technol. Lett 6 (2), 73–78. doi:10.1021/acs.estlett.8b00700

[R198] Shaid A, Wang L, and Padhye R (2018). “Textiles for firefighting protective clothing,” in Firefighters’ clothing and equipment (Florida, United States: CRC Press), 1–30.

[R199] Shane HL, Baur R, Lukomska E, Weatherly L, and Anderson SE (2020). Immunotoxicity and allergenic potential induced by topical application of perfluorooctanoic acid (PFOA) in a murine model. Food Chem. Toxicol 136, 111114. doi:10.1016/j.fct.2020.111114 31904477 PMC7753950

[R200] Shaw SD, Berger ML, Harris JH, Yun SH, Wu Q, Liao C, (2013). Persistent organic pollutants including polychlorinated and polybrominated dibenzo-p-dioxins and dibenzofurans in firefighters from Northern California. Chemosphere 91 (10), 1386–1394. doi:10.1016/j.chemosphere.2012.12.070 23395527

[R201] Shen B, Whitehead TP, McNeel S, Brown FR, Dhaliwal J, Das R, (2015). High levels of polybrominated diphenyl ethers in vacuum cleaner dust from California fire stations. Environ. Sci. Technol 49 (8), 4988–4994. doi:10.1021/es505463g 25798547

[R202] Shibuichi S, Yamamoto T, Onda T, and Tsujii K (1998). Super water- and oil-repellent surfaces resulting from fractal structure. J. Colloid Interface Sci 208 (1), 287–294. doi:10.1006/jcis.1998.5813 9820776

[R203] Shinde A, and Ormond RB (2021). Headspace sampling-gas chromatograph-mass spectrometer as a screening method to thermally extract fireground contaminants from retired firefighting turnout jackets. Fire Mater. 45 (3), 415–428. doi:10.1002/fam.2887 38077743 PMC10698688

[R204] Shoeib M, Harner T, Webster GM, and Lee SC (2011). Indoor sources of poly- and perfluorinated compounds (PFCS) in vancouver, Canada: Implications for human exposure. Environ. Sci. Technol 45 (19), 7999–8005. doi:10.1021/es103562v 21332198

[R205] Shoeib M, Harner T, Wilford BH, Jones KC, and Zhu J (2005). Perfluorinated sulfonamides in indoor and outdoor air and indoor dust: Occurrence, partitioning, and human exposure. Environ. Sci. Technol 39 (17), 6599–6606. doi:10.1021/es048340y 16190217

[R206] Silver G, Krastev Y, Forbes MK, Hamdorf B, Lewis B, Tisbury M, (2021). Study protocol for a randomised controlled trial examining the effect of blood and plasma donation on serum perfluoroalkyl and polyfluoroalkyl substance (PFAS) levels in firefighters. BMJ open 11 (5), e044833. doi:10.1136/bmjopen-2020-044833 PMC8108666 33963058

[R207] Simons J (1949). Production of fluorocarbons. Electrochem. Soc. J 95, 47. doi:10.1149/1.2776733

[R208] Sjogren P, Montse R, Lampa E, Salihovic S, van Bavel B, Lind L, (2016). Circulating levels of perfluoroalkyl substances are associated with dietary patterns – a cross sectional study in elderly Swedish men and women. Environ. Res 150, 59–65. doi:10.1016/j.envres.2016.05.016 27239709

[R209] Smart B (1994). in Characteristics of C-F systems in organofluorine chemistry: Principles and commercial applications. Editors Banks RE, Smart BE, and Tatlow C (New York: Plenum Press).

[R210] Song G, and Lu Y (2013). “Flame resistant textiles for structural and proximity fire fighting,” in Handbook of fire resistant textiles (Amsterdam, Netherlands: Elsevier), 520–548.

[R211] Soteriades ES, Kim J, Christophi CA, and Kales SN (2019). Cancer incidence and mortality in firefighters: A state-of-the-art review and meta-analysis. Asian Pac. J. Cancer Prev. APJCP 20 (11), 3221–3231. doi:10.31557/apjcp.2019.20.11.3221 31759344 PMC7063017

[R212] Stapleton HM, Eagle S, Sjödin A, and Webster TF (2012). Serum PBDEs in a North Carolina toddler cohort: Associations with handwipes, house dust, and socioeconomic variables. Environ. health Perspect 120 (7), 1049–1054. doi:10.1289/ehp.1104802 22763040 PMC3404669

[R213] Steenland K, Fletcher T, Stein CR, Bartell SM, Darrow L, Lopez-Espinosa M-J, (2020). Review: Evolution of evidence on PFOA and health following the assessments of the C8 Science Panel. Environ. Int 145, 106125. doi:10.1016/j.envint.2020.106125 32950793

[R214] Steenland K, Tinker S, Frisbee S, Ducatman A, and Vaccarino V (2009). Association of perfluorooctanoic acid and perfluorooctane sulfonate with serum lipids among adults living near a chemical plant. Am. J. Epidemiol 170 (10), 1268–1278. doi:10.1093/aje/kwp279 19846564

[R215] Steenland K, and Winquist A (2021). PFAS and cancer, a scoping review of the epidemiologic evidence. Environ. Res 194, 110690. doi:10.1016/j.envres.2020.110690 33385391 PMC7946751

[R216] Substances, A. f. T., and Registry, D. (2021). Toxicological profile for perfluoroalkyls. Atlanta, GA: US department of health and human services. Public Health Serv.

[R217] Sunderland EM, Hu XC, Dassuncao C, Tokranov AK, Wagner CC, and Allen JG (2019). A review of the pathways of human exposure to poly- and perfluoroalkyl substances (PFASs) and present understanding of health effects. J. Expo. Sci. Environ. Epidemiol 29 (2), 131–147. doi:10.1038/s41370-018-0094-1 30470793 PMC6380916

[R218] Tansel B (2022). PFAS use in electronic products and exposure risks during handling and processing of e-waste: A review. J. Environ. Manag 316, 115291. doi:10.1016/j.jenvman.2022.115291 35584593

[R219] Tao L, Kannan K, Aldous KM, Mauer MP, and Eadon GA (2008). Biomonitoring of perfluorochemicals in plasma of New York State personnel responding to the World Trade Center disaster. Environ. Sci. Technol 42 (9), 3472–3478. doi:10.1021/es8000079 18522136

[R220] Taylor CK (1999). Fluorinated surfactants in practice. Annu. Surfactants Rev 2, 271–316.

[R221] Tefera YM, Gaskin S, Mitchell K, Springer D, Mills S, and Pisaniello D (2022). Food grown on fire stations as a potential pathway for firefighters’ exposure to per-and poly-fluoroalkyl substances (PFAS). Environ. Int 168, 107455. doi:10.1016/j.envint.2022.107455 35964536

[R222] Temkin AM, Hocevar BA, Andrews DQ, Naidenko OV, and Kamendulis LM (2020). Application of the key characteristics of carcinogens to per and polyfluoroalkyl substances. Int. J. Environ. Res. public health 17 (5), 1668. doi:10.3390/ijerph17051668 32143379 PMC7084585

[R223] Tian M, Peng S, Martin FL, Zhang J, Liu L, Wang Z, (2012). Perfluorooctanoic acid induces gene promoter hypermethylation of glutathione-S-transferase Pi in human liver L02 cells. Toxicology 296 (1-3), 48–55. doi:10.1016/j.tox.2012.03.003 22425687

[R224] Tonnelier A, Coecke S, and Zaldívar J-M (2012). Screening of chemicals for human bioaccumulative potential with a physiologically based toxicokinetic model. Archives Toxicol. 86 (3), 393–403. doi:10.1007/s00204-011-0768-0 PMC3282909 22089525

[R225] Trowbridge J, Gerona RR, Lin T, Rudel RA, Bessonneau V, Buren H, (2020). Exposure to perfluoroalkyl substances in a cohort of women firefighters and office workers in San Francisco. Environ. Sci. Technol 54 (6), 3363–3374. doi:10.1021/acs.est.9b05490 32100527 PMC7244264

[R226] Trudel D, Horowitz L, Wormuth M, Scheringer M, Cousins IT, and Hungerbühler K (2008). Estimating consumer exposure to PFOS and PFOA. Risk Analysis An Int. J 28 (2), 251–269. doi:10.1111/j.1539-6924.2008.01017.x 18419647

[R227] van der Veen I, Hanning A-C, Stare A, Leonards PEG, de Boer J, and Weiss JM (2020). The effect of weathering on per- and polyfluoroalkyl substances (PFASs) from durable water repellent (DWR) clothing. Chemosphere 249, 126100. doi:10.1016/j.chemosphere.2020.126100 32062207

[R228] van der Veen I, Schellenberger S, Hanning A-C, Stare A, de Boer J, Weiss JM, (2022). Fate of per-and polyfluoroalkyl substances from durable water-repellent clothing during use. Washington, DC: Environmental science & technology. 10.1021/acs.est.1c07876 PMC9069696 35404577

[R229] Vaughn B, Andrea W, and Kyle S (2013). Perfluorooctanoic acid (PFOA) exposures and incident cancers among adults living near a chemical plant. Environ. health Perspect 121 (11-12), 1313–1318. doi:10.1289/ehp.1306615 24007715 PMC3855514

[R230] Vieira Verónica M, Kate H, Shin H-M, Weinberg Janice M, Webster Thomas F, and Tony F (2013). Perfluorooctanoic acid exposure and cancer outcomes in a contaminated community: A geographic analysis. Environ. health Perspect 121 (3), 318–323. doi:10.1289/ehp.1205829 23308854 PMC3621179

[R231] von Ehrenstein OS, Fenton SE, Kato K, Kuklenyik Z, Calafat AM, and Hines EP (2009). Polyfluoroalkyl chemicals in the serum and milk of breastfeeding women. Reprod. Toxicol 27 (3-4), 239–245. doi:10.1016/j.reprotox.2009.03.001 19429402

[R232] Wang F, Liu W, Ma J, Yu M, Jin Y, and Dai J (2012). Prenatal and neonatal exposure to perfluorooctane sulfonic acid results in changes in miRNA expression profiles and synapse associated proteins in developing rat brains. Environ. Sci. Technol 46 (12), 6822–6829. doi:10.1021/es3008547 22594572

[R233] Wang H, Du H, Yang J, Jiang H, Karmin O, Xu L, (2019). PFOS, PFOA, estrogen homeostasis, and birth size in Chinese infants. Chemosphere 221, 349–355. doi:10.1016/j.chemosphere.2019.01.061 30641376

[R234] Wang J, Mao G, Ober CK, and Kramer EJ (1997). Liquid crystalline, semifluorinated side group block copolymers with stable low energy surfaces: Synthesis, liquid crystalline structure, and critical surface tension. Macromolecules 30 (7), 1906–1914. doi:10.1021/ma961412o

[R235] Wang Y, Zhang L, Teng Y, Zhang J, Yang L, Li J, (2018). Association of serum levels of perfluoroalkyl substances with gestational diabetes mellitus and postpartum blood glucose. J. Environ. Sci 69, 5–11. doi:10.1016/j.jes.2018.03.016 29941268

[R236] Wang Z, Cousins IT, Scheringer M, Buck RC, and Hungerbühler K (2014). Global emission inventories for C4–C14 perfluoroalkyl carboxylic acid (PFCA) homologues from 1951 to 2030, part II: The remaining pieces of the puzzle. Environ. Int 69, 166–176. doi:10.1016/j.envint.2014.04.006 24861268

[R237] Wang Z, DeWitt JC, Higgins CP, and Cousins IT (2017). A never-ending story of per-and polyfluoroalkyl substances (PFASs)?. Washington, DC: Environmental Science & Technology. 10.1021/acs.est.6b04806 28224793

[R238] Washington JW, and Jenkins TM (2015). Abiotic hydrolysis of fluorotelomer-based polymers as a source of perfluorocarboxylates at the global scale. Environ. Sci. Technol 49 (24), 14129–14135. doi:10.1021/acs.est.5b03686 26526296

[R239] Washington JW, Jenkins TM, Rankin K, and Naile JE (2015). Decades-scale degradation of commercial, side-chain, fluorotelomer-based polymers in soils and water. Environ. Sci. Technol 49 (2), 915–923. doi:10.1021/es504347u 25426868

[R240] Water ZD (2012). Durable Water and Soil Repellent Chemistry in the Textile Industry - A Research Report *[Online]*. Available at: https://uploads-ssl.webflow.com/5c4065f2d6b53e08a1b03de7/5db6eece578efb688bee2bed_DWR_Report.pdf (Accessed December 15, 2022).

[R241] Weatherly LM, Shane HL, Lukomska E, Baur R, and Anderson SE (2021). Systemic toxicity induced by topical application of heptafluorobutyric acid (PFBA) in a murine model. Food Chem. Toxicol 156, 112528. doi:10.1016/j.fct.2021.112528 34474067 PMC8693634

[R242] Wielsøe M, Long M, Ghisari M, and Bonefeld-Jørgensen EC (2015). Perfluoroalkylated substances (PFAS) affect oxidative stress biomarkers *in vitro*. Chemosphere 129, 239–245. doi:10.1016/j.chemosphere.2014.10.014 25455676

[R243] Wikström S, Lin P-I, Lindh CH, Shu H, and Bornehag C-G (2020). Maternal serum levels of perfluoroalkyl substances in early pregnancy and offspring birth weight. Pediatr. Res 87 (6), 1093–1099. doi:10.1038/s41390-019-0720-1 31835271 PMC7196936

[R244] Winkens K, Koponen J, Schuster J, Shoeib M, Vestergren R, Berger U, (2017). Perfluoroalkyl acids and their precursors in indoor air sampled in children’s bedrooms. Environ. Pollut 222, 423–432. doi:10.1016/j.envpol.2016.12.010 28012670

[R245] Wong F, MacLeod M, Mueller JF, and Cousins IT (2014). Enhanced elimination of perfluorooctane sulfonic acid by menstruating women: Evidence from population-based pharmacokinetic modeling. Environ. Sci. Technol 48 (15), 8807–8814. doi:10.1021/es500796y 24943117

[R246] Xu Y, Nielsen C, Li Y, Hammarstrand S, Andersson EM, Li H, (2021). Serum perfluoroalkyl substances in residents following long-term drinking water contamination from firefighting foam in Ronneby, Sweden. Environ. Int 147, 106333. doi:10.1016/j.envint.2020.106333 33360412

[R247] Young AS, Sparer-Fine EH, Pickard HM, Sunderland EM, Peaslee GF, and Allen JG (2021). Per-and polyfluoroalkyl substances (PFAS) and total fluorine in fire station dust. J. Expo. Sci. Environ. Epidemiol 31 (5), 930–942. doi:10.1038/s41370-021-00288-7 33542478 PMC8339150

[R248] Yu N, Wei S, Li M, Yang J, Li K, Jin L, (2016). Effects of perfluorooctanoic acid on metabolic profiles in brain and liver of mouse revealed by a high-throughput targeted metabolomics approach. Sci. Rep 6 (1), 23963. doi:10.1038/srep23963 27032815 PMC4817033

[R249] Zhao P, Xia X, Dong J, Xia N, Jiang X, Li Y, (2016). Short-and long-chain perfluoroalkyl substances in the water, suspended particulate matter, and surface sediment of a turbid river. Sci. Total Environ 568, 57–65. doi:10.1016/j.scitotenv.2016.05.221 27285797

[R250] Zhou J, Jenkins TG, Jung AM, Jeong KS, Zhai J, Jacobs ET, (2019). DNA methylation among firefighters. PLoS One 14 (3), e0214282. doi:10.1371/journal.pone.0214282 30913233 PMC6435149

